# On Tractable Convex Relaxations of Standard Quadratic Optimization Problems under Sparsity Constraints

**DOI:** 10.1007/s10957-024-02593-1

**Published:** 2025-01-23

**Authors:** Immanuel Bomze, Bo Peng, Yuzhou Qiu, E. Alper Yıldırım

**Affiliations:** 1 Faculty of Mathematics and Research Network Data Science, University of Vienna, Oskar-Morgenstern-Platz 1, 1090 Wien, Austria; 2 VGSCO and ISOR, University of Vienna, Oskar-Morgenstern-Platz 1, 1090 Wien, Austria; 3 School of Mathematics, The University of Edinburgh, Peter Guthrie Tait Road, Edinburgh, EH9 3FD UK

**Keywords:** Standard quadratic optimization problems, Sparsity, Mixed-integer quadratic optimization, Reformulation-linearization technique, Shor relaxation, 90C11, 90C20, 90C22

## Abstract

Standard quadratic optimization problems (StQPs) provide a versatile modelling tool in various applications. In this paper, we consider StQPs with a hard sparsity constraint, referred to as sparse StQPs. We focus on various tractable convex relaxations of sparse StQPs arising from a mixed-binary quadratic formulation, namely, the linear optimization relaxation given by the reformulation–linearization technique, the Shor relaxation, and the relaxation resulting from their combination. We establish several structural properties of these relaxations in relation to the corresponding relaxations of StQPs without any sparsity constraints, and pay particular attention to the rank-one feasible solutions retained by these relaxations. We then utilize these relations to establish several results about the quality of the lower bounds arising from different relaxations. We also present several conditions that ensure the exactness of each relaxation.

## Introduction

The Standard Quadratic optimization Problem (StQP) consists of minimizing a quadratic form over the standard simplex (all vectors with no negative coordinates that sum up to one).

If the quadratic form is convex or concave, StQP can be solved in polynomial time. However, since the maximum-clique problem admits a reformulation in the form of StQP [34], this problem class is NP-hard. Therefore, we can view the class of StQP as the simplest of the hard problems: the simplest nonconvex objective functions are generated by indefinite Hessians, and the feasible set is the simplest bounded polyhedron (polytope) with a very obvious structure of faces comprised of standard simplices in lower dimensions when some variables are fixed to zero.

Despite its simplicity, the class of StQPs provides a quite versatile modelling tool (see, e.g., [2]). Applications are numerous, ranging from the famous Markowitz portfolio problem in finance [31, 32], evolutionary game theory in economics  [7, 37, 41] and quadratic resource allocation problems  [24], through machine learning (background-foreground clustering in image analysis and pattern recognition [36]), to the life sciences — e.g., in population genetics (selection models, [27]) and ecology/evolutionary population (replicator) dynamics [22, 23]. For an overarching review combining dynamic modelling, optimization, and game theory, the interested reader may refer to [3].

StQPs appear also quite naturally as subproblems in copositive-conic relaxations of mixed-integer or combinatorial optimization problems of all sorts. Finally, using barycentric coordinates, every quadratic optimization problem over a polytope with known vertices can be rephrased as an instance of (potentially exponential-sized) StQP.

The aforementioned structural simplicity does not preclude coexistence of an exponential number of (local or global) solutions to some StQPs. Some of these solutions may be sparse (and will be so with high probability in the average case, see below), others may have many positive coordinates. However, in important applications like some variants of sparse portfolio optimization problems where one is interested in investments with a limited number of assets (see, e.g., [33] and the references therein; many empirical and theoretical studies in this domain, e.g., [1, 16, 18] corroborate significance of this approach, which seems also to alleviate some estimation problems of the risk structure), sparsity of a solution must be enforced by an additional, explicit hard constraint on the number of positive coordinates. Introducing this sparsity constraint can render StQPs NP-hard even if the Hessian is positive-definite.

This paper deals with such problems and investigates the structural properties of tractable linear and semidefinite relaxations which scale well with the dimension.

## Background, Motivation, and Layout of Contribution

In this section, we provide some background on standard quadratic optimization problems. We present our motivation for studying the variant with a hard sparsity constraint. We introduce our notation and give an outline of the paper.

### The Combinatorial Nature of Standard Quadratic Optimization – Coexistence of Solutions and Role of Active Sets

The well-studied Standard Quadratic optimization Problem (StQP) is given by 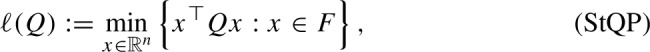


where $Q \in \mathcal{S}^n$ is the problem data and $\mathcal{S}^n$ denotes the space of $n \times n$ real symmetric matrices, $x \in \mathbb {R}^n$ is the decision variable, and $F \subset \mathbb {R}^n$ denotes the standard simplex given by

1 $$\begin{aligned} F:= \left\{ x \in \mathbb {R}^n: e^\top x = 1, \quad x \ge 0\right\} , \end{aligned}$$

where $e \in \mathbb {R}^n$ denotes the vector of all ones.

There is an exponential number, namely $2^n-1$, of faces of *F*, which form the “combinatorial” reason for NP-hardness (see Proposition 2.1). Indeed, if the active set $\{ i: x_i^* =0 \}$ at the global solution $x^*$ is known exactly, locating the solution (i.e., determining $x^*$ or a value-equivalent alternative with the same set of zero coordinates) reduces to solving an $n\times n$ linear equation system. The same holds true for locating local solutions and even first-order critical (KKT) points. This phenomenon may be the reason why recently iterative first-order methods were proposed, which can achieve identification of the correct active set in finite time [10].

For any instance of (StQP), not all faces of *F* can contain an isolated (local or global) solution in their relative interior, as there is an upper bound on their cardinality given by Sperner’s theorem on the maximal antichain (and Stirling’s asymptotics), namely

2 $$\begin{aligned} \left( {\begin{array}{c}n\\ \lfloor \frac{n}{2}\rfloor \end{array}}\right) \sim \sqrt{\frac{2}{\pi n}}\, 2^n\quad \text{ as } n\rightarrow \infty \,. \end{aligned}$$

Scozzari and Tardella [38] show that solutions can occur only in the relative interior of a face restricted to which the objective function is strictly convex. Nevertheless, recent research [11] has shown an exponential behavior regarding the number of local (or global) solutions: in the worst case, an instance of (StQP) of order *n* can have at least

3 $$\begin{aligned} (15120)^{n/{24}} \approx (1.4933)^n \end{aligned}$$

coexisting optimal solutions, a lower bound that currently seems to be the largest one known. The other bad news is that rounding on the standard simplex is, from the asymptotic point of view, also not always successful [5]. In spite of all this, (StQP) admits a polynomial-time approximation scheme (PTAS) [4].

### Worst-case versus Average-Case Behavior–Expected Sparsity

All of the above observations refer to the worst case, of course. Several researchers turned to the average case, modelled by randomly chosen instances. Already in 1988, Kingman  [26] observed that very large polymorphisms (i.e., solutions $x^*$ with more than $C\sqrt{n}$ positive coordinates where *C* is a positive constant independent of *n*) are atypical. More recently, in a series of papers Kontogiannis and Spirakis [28–30] looked at models with several independent and identically distributed (e.g., Gaussian or uniform) entries of $Q \in \mathcal{S}^n$ and proved, among other results, that the expected number of (local) solutions does not grow faster than $\exp (0.138n)\approx (1.148)^n$, way smaller than the worst-case lower bound in (3). Based upon more recent research by Chen and coauthors [14, 15], under quite reasonable distributional assumptions modeling the random average case, the probability that the global solution has more than two positive coordinates (i.e., that it does not lie on an edge of *F*) is asymptotically vanishing faster than

$$\begin{aligned} K \frac{(\log n)^2}{n}\quad \text{ with } n\rightarrow \infty , \end{aligned}$$

where $K>0$ is a universal constant [11, Proposition 1].

### StQPs with a Hard Sparsity Constraint

However, if the instances are somehow structured, we cannot rely on our “luck” that *Q* exhibits an average behavior in the above sense, and still, we may prefer a sparse solution to (StQP). So, in pursuit of these sparse solutions, we introduce the following variant under a cardinality constraint, referred to as the *sparse StQP*:

4 $$\begin{aligned} \ell _\rho (Q):= \min \limits _{x \in \mathbb {R}^n} \left\{ x^\top Q x: x \in F_\rho \right\} , \end{aligned}$$

where

5 $$\begin{aligned} F_\rho := \left\{ x \in F: \quad \Vert x\Vert _0 \le \rho \right\} \,. \end{aligned}$$

Here, $\Vert x\Vert _0$ denotes the number of nonzero components of a vector *x* and $\rho \in \{1,\ldots ,n\}$ is the sparsity parameter.

The elements of $F_\rho$ will be referred to as $\rho$-sparse. Evidently, any (feasible or optimal) solution of the sparse StQP is a feasible solution to (StQP) with guaranteed $\rho$-sparsity, which can be crucial.

We start with some simple observations.

#### Lemma 2.1

The following relations hold:

6 $$\begin{aligned} \ell (Q) = \ell _n(Q) \le \ell _{n-1}(Q) \le \ldots \le \ell _2(Q)\le \ell _1(Q) \,, \end{aligned}$$

with

7 $$\begin{aligned} \ell _1(Q) = \min \limits _{1\le k \le n} Q_{kk} \,, \end{aligned}$$

and

8 $$\begin{aligned} \ell _2(Q) = \min \left\{ \min \left\{ \textstyle {\frac{Q_{ii}Q_{jj}-Q_{ij}^2}{Q_{ii}+Q_{jj}-2Q_{ij}}}: Q_{ij}< \min \{Q_{ii},Q_{jj}\}, {1\le i<j\le n} \right\} , \, \ell _1(Q)\right\} \,.\nonumber \\ \end{aligned}$$

Furthermore, we have $\ell (Q) = \ell _\rho (Q)$ if and only if (StQP) has a $\rho$-sparse optimal solution.

#### Proof

The relations (6) and (7) follow from $F_1 = \{e^1,e^2,\ldots ,e^n\} \subset F_2 \subset \cdots \subset F_{n-1} \subset F_n = F$, where $F_\rho$ and *F* are given by (1) and (5), respectively, and $e^i \in \mathbb {R}^n$ denotes the *i*th unit vector, $i = 1,\ldots ,n$. For $\rho =2$, a straightforward discussion of univariate quadratics over the edges ${{\,\textrm{conv}\,}}\left( \left\{ e^i,e^j\right\} \right) ,~1 \le i < j \le n$ (in case these are strictly convex functions yielding a minimizer in the relative interior of the edge) is sufficient to establish (8). The last assertion is trivial. $\square$

The condition $\ell (Q)=\ell _2(Q)$ is related to edge-convexity of the instance of (StQP) as discussed in [38, Theorem 1] but we will not dive into details here. Rather observe that the effort to calculate $\ell _2(Q)$, obviously always an upper bound of $\ell (Q)$, is the same as for the closed-form lower bound $\ell ^\textrm{ref}(Q)\le \ell (Q)$ proposed in [6]. The lower bound $\ell ^\textrm{ref}(Q)$ of the bracket $\ell ^\textrm{ref}(Q)\le \ell (Q) \le \ell _2(Q)$ is exact if and only if all off-diagonal entries of *Q* are equal [6, Theorem 2], and the optimal solution $x^*$ to (StQP) can have any $\Vert x^*\Vert _0\le n$. By contrast, as argued above, the upper bound $\ell _2(Q)$ is exact if and only if an optimal solution $x^*$ to (StQP) satisfies $\Vert x^*\Vert _0 \le 2$.

### Worst-Case Complexity and Polynomial Approximability of the Sparse StQP

The set $F_\rho$ is the union of $$\left( {\begin{array}{c}n\\ \rho \end{array}}\right) = {\mathcal {O}} (n^\rho )$$ faces of *F*, a number polynomial in *n*. In each of these faces, due to (2), at most $$\left( {\begin{array}{c}\rho \\ \lfloor \frac{\rho }{2}\rfloor \end{array}}\right) $$ local solutions to (4) can coexist. If $\rho$ is fixed independently of *n*, we hence end up with a polynomial set of candidates which makes problem (4) solvable in polynomial time. However, even if $\rho$ is fixed to a moderate number, say to 6, and for medium-scale dimensions, say $n=100$, polynomial worst-case behavior would not help much in practical optimization since $n^\rho =10^{12}$. This emphasizes the need for tractable relaxations of the sparse StQP.

On the other hand, if $\rho \rightarrow \infty$ and also $n-\rho \rightarrow \infty$ like in the case of $\rho = \lfloor \gamma n\rfloor$ for some $\gamma$ with $0<\gamma <1$, then Stirling’s asymptotics show an exponential increase for $$\left( {\begin{array}{c}n\\ \rho \end{array}}\right) $$, e.g.

9 $$\begin{aligned} \left( {\begin{array}{c}n\\ \lfloor \gamma n \rfloor \end{array}}\right) \sim \frac{1}{\sqrt{2\pi \gamma (1-\gamma )n}}\, \left[ \gamma ^{-\gamma }(1-\gamma )^{\gamma -1} \right] ^n \quad \text{ as } n\rightarrow \infty \,. \end{aligned}$$

More generally speaking, if $0< \gamma \le \frac{\rho }{n} \le \delta <1$, then $$\left( {\begin{array}{c}n\\ \rho \end{array}}\right) \ge K\left[ \delta ^{-\gamma }(1-\gamma )^{\delta -1}\right] ^n$$, while in case $\frac{\rho }{n} \rightarrow 0$ but $\rho \rightarrow \infty$ the term $\left( \frac{ne}{\rho }\right) ^\rho /\sqrt{\rho }$ is dominant, neither of which can be bounded by a polynomial in *n*.

Above observations suggest that the sparse StQP is NP-hard even when *Q* is positive-definite due to the combinatorial nature of the sparsity term $\Vert x\Vert _0$. We will prove this in the following.

#### Proposition 2.1

If $(n,\rho )$ are considered as input, the sparse StQP is NP-hard even if *Q* is positive-definite.

#### Proof

Our arguments are similar to those in [20] who dealt with (sign-unconstrained) sparse portfolio selection. In the online supplement to [20], this problem is reduced to the *k*-subset sum problem by the following technique: consider the following instance of the decision version of the latter problem. Given *n* integers $\{a_1,\ldots ,a_n\}$ and $\rho$, decide whether or not there is a $\rho$-element subset $S\subset \{ 1,\ldots , n\}$ with $\sum _{i\in S} a_i=0$. This amounts to finding a binary vector $v\in \{0,1\}^n$ such that ${\Vert v \Vert }_0=\rho$ and $a^\top v= 0$, which, in turn, is equivalent to $v^\top H v =0$ where $H=aa^\top$, or else certifying that no such *v* exists. To this end, Gao and Li [20] consider the minimization of the sum of a squared Euclidean distance and $v^\top H v$ over $\mathbb {R}^n$ subject to the constraint ${\Vert v \Vert }_0 \le \rho$. We do basically the same but scale it, and additionally pose sign constraints: consider the quadratic objective $q(x)= \Vert x-\frac{1}{\rho }\, e \Vert _2^2 +x^\top H x = x^\top (H + I) x - \frac{2}{\rho } e^\top x + n$ and minimize it over $x\in F_\rho$. Over this set, we have

$$\begin{aligned}{ \left\| x- \textstyle \frac{1}{\rho }\, e\right\| _2^2 = \sum \limits _{j: x_j > 0} \left( x_j - \textstyle \frac{1}{\rho }\right) ^2 + \sum \limits _{j: x_j = 0} \textstyle \frac{1}{\rho ^2} \ge \frac{n-\rho }{\rho ^2}\,,} \end{aligned}$$

where we used $\Vert x \Vert _0 \le \rho$ to derive the inequality. Furthermore, this holds with equality if and only if $v:=\rho \, x$ is a binary vector. Putting $Q=\frac{1}{2}\,\nabla ^2 q = H + I \succ 0$, we therefore obtain $\ell _\rho (Q) \ge \frac{n-\rho }{\rho ^2} + \frac{2}{\rho } - n$ for this problem. If this inequality is strict, we have a certificate of unsolvability of the *k*-subset sum problem. Else if $\ell _\rho (Q)= \frac{n-\rho }{\rho ^2} + \frac{2}{\rho } - n$, then necessarily *v* is binary and satisfies $a^\top v=0$. Hence solving for $\ell _\rho (Q)$ cannot be easier than solving the *k*-subset sum problem, which is NP-complete. This proves the claim. $\square$

As we have seen above, for bounded $\rho$ while $n\rightarrow \infty$, the sparse StQP remains polynomially solvable even for indefinite *Q*. Note that, on the other extreme case of sparsity/density, where $n-\rho \le \beta$ with a fixed integer $\beta >0$, we still get a polynomial number of faces as $$\left( {\begin{array}{c}n\\ \rho \end{array}}\right) =\left( {\begin{array}{c}n\\ n-\rho \end{array}}\right) = {\mathcal {O}}(n^\beta )$$, but the dimensions of the faces now increase linearly with *n*, which still maintains NP-hardness in the non-convex case. However, in this case we can at least transfer an approximability result from the StQP without sparsity:

#### Proposition 2.2

If $\beta$ is a constant, then the sparse StQP with $n-\rho \le \beta$ allows for a PTAS, even if *Q* is indefinite.

#### Proof

On every face of $F_\rho$, we have to solve an StQP with at most $n-\rho <n$ variables, which all allow for a PTAS, see [4, Thm.3.2]. But there are at most ${\mathcal {O}}(n^\beta )$ such faces, which proves the claim by cumulation. $\square$

The approximability status of the general sparse StQP remains unclear, e.g., in the cases considered in (9); note that we cannot use the same reduction technique as above to infer from approximability of the subset-sum problem [25] a similar approximability result for our sparse StQP. Indeed, in general, approximability results do not necessarily translate to reductions.

### Mixed-Binary Quadratic Formulation of Sparse StQPs and Contributions

By introducing binary variables, the sparse StQP can be reformulated as a mixed-binary QP: 
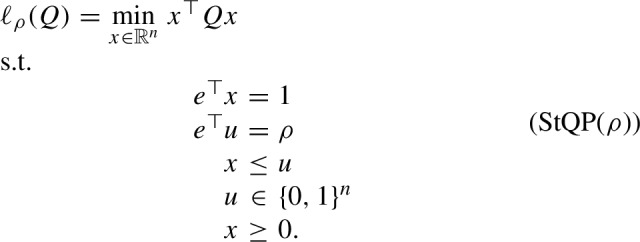


In this paper, we focus on various convex relaxations of (StQP($\rho$)), all more tractable than the conic ones presented in [8, Section 3.2] for general quadratic optimization problems. In particular, we establish several structural properties of these relaxations and shed light on the relations between each relaxation of (StQP($\rho$)) and the corresponding relaxation of (StQP). We then draw several conclusions about the relations between different relaxations as well as the strength of each relaxation.

While it turns out that all relaxations behave as expected for the case of $\rho = n$, already for the cases $\rho =1$ and $\rho =2$ (which cannot be excluded with a high probability in the random average case models) and other moderate sparsity values, there is a sharp contrast between the relaxations, which contributes to the motivation of this study. Typically, applications would require models with sparsity (significantly) less than half of the dimension, for which we obtain more interesting results.

We will also pay particular attention to the case of rank-one solutions to the relaxations (all of them use matrix variables by lifting), in particular, because they certify optimality if optimal to the relaxed problems, and also because in algorithmic frameworks, we may (warm-)start with some (good) feasible solutions to the original problem of larger sparsity than desired.

After the first version of this manuscript was submitted, the authors continued to investigate alternative convex relaxations of the sparse StQP arising from copositive reformulations of two mixed-binary quadratic models [9], one of which is given by (StQP($\rho$)). They studied tighter but computationally more expensive doubly nonnegative relaxations and established novel reformulations of these relaxations in smaller dimensions. In contrast, our goal in this paper is to shed light on the structural properties of cheaper classical relaxations, especially in comparison with the corresponding relaxation without the sparsity constraint, and to understand their strengths and limitations.

### Notation and Organisation of the Paper

We use $\mathbb {R}^n$, $\mathbb {R}^n_+$, $\mathbb {R}^{m \times n}$, and $\mathcal{S}^n$ to denote the *n*-dimensional Euclidean space, the nonnegative orthant, the set of $m \times n$ real matrices, and the space of $n \times n$ real symmetric matrices, respectively. We use 0 to denote the real number 0, the vector of all zeroes, as well as the matrix of all zeroes, which should always be clear from the context. We denote by $e \in \mathbb {R}^n$, $e^i \in \mathbb {R}^n,~1 \le i \le n$, $I \in \mathcal{S}^n$, and $E = ee^\top \in \mathcal{S}^n$ the vector of all ones, the *i*th unit vector, the identity matrix, and the matrix of all ones, respectively. All inequalities on vectors or matrices are understood to be applied componentwise. For $A \in \mathcal{S}^n$ and $B \in \mathcal{S}^n$, we use $A \succeq B$ to denote that $A - B$ is positive-semidefinite. We use $x_j$ and $A_{ij}$ to denote the components of $x \in \mathbb {R}^n$ and $A \in \mathbb {R}^{m \times n}$. For $B \in \mathbb {R}^{n \times n}$ and $b \in \mathbb {R}^n$, we denote by ${{\,\textrm{diag}\,}}(B) \in \mathbb {R}^n$ and ${{\,\textrm{Diag}\,}}(b) \in \mathcal{S}^n$ the vector given by the diagonal entries of *B* and the diagonal matrix whose diagonal entries are given by *b*, respectively. The convex hull of a set is denoted by ${{\,\textrm{conv}\,}}(\cdot )$. For any $u \in \mathbb {R}^n$ and $v \in \mathbb {R}^n$, $u^\top v$ denotes the Euclidean inner product. Similarly, for any $U \in \mathbb {R}^{m \times n}$ and $V \in \mathbb {R}^{m \times n}$, the trace inner product is denoted by

$$\begin{aligned} \langle U, V \rangle = \text {trace}(U{^\top } V) = \sum \limits _{i=1}^m \sum \limits _{j = 1}^n U_{ij} V_{ij}. \end{aligned}$$

The paper is organized as follows. In Sect. 3, we consider several convex relaxations of (StQP($\rho$)). Section 3.1 focuses on the RLT (reformulation–linearization technique) relaxation of (StQP($\rho$)) and presents several results in comparison with the RLT relaxation of (StQP). The Shor relaxation of (StQP($\rho$)) is treated in Sect. 3.2 and compared with that of (StQP). In Sect. 4, we then study the convex relaxation of (StQP($\rho$)) given by combining the RLT and Shor relaxations and compare it with that of (StQP). We conclude the paper in Sect. 5.

## Convex Relaxations: RLT and Shor

In this section, we consider several well-known convex relaxations of (StQP($\rho$)), which use LP (linear programming) and SDP (semidefinite programming) methods. We study their properties and establish relations between each relaxation of (StQP($\rho$)) and the corresponding relaxation of (StQP).

### RLT Relaxation

In this section, we consider the RLT (reformulation–linearization technique) relaxation of (StQP($\rho$)) and compare it with the RLT relaxation of (StQP).

RLT relaxations of optimization problems with a quadratic objective function and a mix of linear and quadratic constraints are obtained by a two-stage process (see, e.g., [39]). The first stage, referred to as reformulation, consists of generating (additional) valid quadratic constraints from linear constraints by multiplying each pair of linear inequality constraints as well as each linear equality constraint by each variable. In the second stage, referred to as linearization, all of the original and additional quadratic functions are linearized by replacing the quadratic terms $x_i x_j$ by a lifted variable $X_{ij},~1 \le i \le j \le n$. Together with the original linear constraints, this gives rise to the RLT relaxation.

We first start with the RLT relaxation of (StQP): 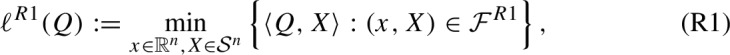 where

10 $$\begin{aligned} \mathcal{F}^{R1}:= \left\{ (x,X) \in \mathbb {R}^n \times \mathcal{S}^n: e^\top x = 1, \quad X e = x, \quad x \ge 0, \quad X \ge 0\right\} . \end{aligned}$$

Note that $x \ge 0$ is a redundant constraint in $\mathcal{F}^{R1}$ since it is implied by $X e = x$ and $X \ge 0$. Furthermore, it is easy to see that $\mathcal{F}^{R1}$ is a polytope. We first recall the following result about (R1).

#### Proposition 3.1

(Qiu and Yıldırım [35]) The set of vertices of $\mathcal{F}^{R1}$ is given by

11 $$\begin{aligned}  &   \left\{ (e^i, e^i (e^i)^\top ): i = 1,\ldots ,n\right\} \nonumber \\  &   \qquad \cup \left\{ \left( \frac{1}{2}(e^i+e^j), \frac{1}{2}(e^i (e^j)^\top + e^j (e^i)^\top )\right) : 1 \le i < j \le n\right\} . \end{aligned}$$

Therefore,

$$\begin{aligned} \ell ^{R1} (Q) = \min \limits _{1\le i \le j \le n} Q_{ij} \le \ell (Q). \end{aligned}$$

Furthermore, (R1) is exact (i.e., $\ell ^{R1} (Q) = \ell (Q)$) if and only if

$$\begin{aligned} \min \limits _{1\le i \le j \le n} Q_{ij} = \min \limits _{1\le k \le n} Q_{kk}. \end{aligned}$$

#### Remark 3.1

As pointed out by a reviewer, Proposition 3.1 can be proved from another perspective. If $(x,X) \in \mathcal{F}^{R1}$, then we clearly have $\langle E, X \rangle = e^\top X e = e^\top x = 1$ and $X \ge 0$. Conversely, whenever $X \in \mathcal{S}^n$ satisfies $\langle E, X \rangle = 1$ and $X \ge 0$, it is easy to see that $(x, X) = (Xe, X) \in \mathcal{F}^{R1}$. Therefore,

12 $$\begin{aligned} \ell ^{R1}(Q) = \min \limits _{X \in \mathcal{S}^n} \left\{ \langle Q, X \rangle : X \in \mathcal{G}^{R1}\right\} , \end{aligned}$$

where $\mathcal{G}^{R1}:= \left\{ X \in \mathcal{S}^n: \langle E, X \rangle = 1, \quad X \ge 0\right\}$. It is easy to verify that (*x*, *X*) is a vertex of $\mathcal{F}^{R1}$ if and only if *X* is a vertex of $\mathcal{G}^{R1}$. Defining $\mu _{ii} = X_{ii},~i = 1,\ldots ,n$, and $\mu _{ij} = X_{ij} + X_{ji} = 2X_{ij},~1 \le i < j \le n$, it follows from (12) that

$$\begin{aligned} \ell ^{R1}(Q)= &   \min \limits _{\mu _{ij} \in \mathbb {R}: 1 \le i \le j \le n} \left\{ \sum \limits _{1 \le i \le j \le n} \mu _{ij} (e^i)^\top Q e^j:\right. \\  &   \left. \sum \limits _{1 \le i \le j \le n} \mu _{ij} = 1, \quad \mu _{ij} \ge 0, \quad 1 \le i \le j \le n\right\} . \end{aligned}$$

Proposition 3.1 now follows from the simple structure of the vertices of the standard simplex in $\mathbb {R}^{n(n+1)/2}$ and the aforementioned transformations. We remark that this is precisely the initial inner approximation of the copositive cone used in the adaptive polyhedral approximation scheme based on simplicial refinements proposed in [12]. The reader is also referred to [19] and [42] for uniform inner and outer polyhedral approximations of the copositive cone, respectively.

Proposition 3.1 implies that (R1) is exact if and only if the minimum entry of *Q* is on the diagonal. In this case, (StQP) has a 1-sparse optimal solution, i.e., the optimal solution of (StQP) without any sparsity constraint is already the sparsest possible solution. Furthermore, by Lemma 2.1, we immediately obtain

13 $$\begin{aligned} \ell (Q) = \ell _n(Q) = \ell _{n-1}(Q) = \ldots = \ell _1(Q) = \min \limits _{1\le k \le n} Q_{kk}. \end{aligned}$$

By reformulating the binarity constraint $u_j \in \{0,1\}$ with $u_j^2 = u_j,~j = 1,\ldots ,n$ in (StQP($\rho$)) and linearizing the quadratic terms $x x^\top , x u^\top$, and $u u^\top$ by *X*, *R*, and *U*, respectively, we obtain the following RLT relaxation: 
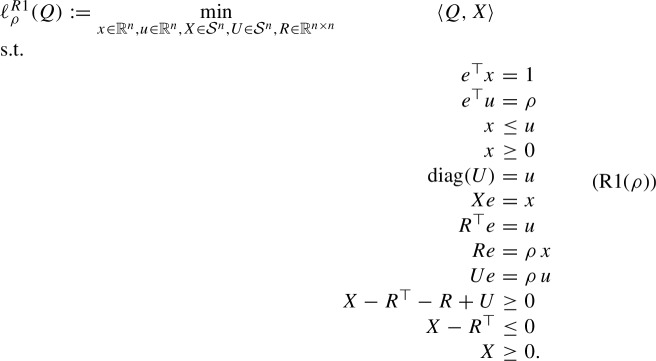


Note that $u\le e$ is *not* implied in this formulation. We next establish some basic properties of (R1($\rho$)).

#### Lemma 3.1

The constraints $x \le u$ and $x \ge 0$ are redundant in (R1($\rho$)). Furthermore, if (*x*, *u*, *X*, *U*, *R*) is R1($\rho$)-feasible, then

$$\begin{aligned} u\ge 0, \quad R \ge 0, \quad R - U \le 0, \quad U \ge 0. \end{aligned}$$

#### Proof

It is easy to see that the constraints $x \le u$ and $x \ge 0$ are implied by the constraints $Xe = x$, $X \ge 0$, $R^\top e = u$, and $X - R^\top \le 0$. Since $u \ge 0$ is a redundant constraint in (StQP($\rho$)), the RLT constraints implied by this constraint are also redundant in (R1($\rho$)) (see, e.g., [40, Proposition 2]). Indeed, it is easy to see that the RLT constraints $R \ge 0$, $R - U \le 0$, and $U \ge 0$ are implied by $X \ge 0$, $X - R^\top \le 0$, and $X - R^\top - R + U\ge 0$. $\square$

Let us denote the projection of the feasible region of (R1($\rho$)) onto (*x*, *X*) by

14 $$\begin{aligned} \mathcal{F}^{R1}_\rho:= &   \left\{ (x,X) \in \mathbb {R}^n \times \mathcal{S}^n: (x,u,X,U,R) \text { is } (R1(\rho )) \text {-feasible for some }\right. \nonumber \\  &   \left. (u,U,R) \in \mathbb {R}^n \times \mathcal{S}^n \times \mathbb {R}^{n \times n}\right\} \,. \end{aligned}$$

Note that

15 $$\begin{aligned} \ell ^{R1}_\rho (Q) = \min \limits _{(x,X) \in \mathbb {R}^n \times \mathcal{S}^n}\left\{ \langle Q, X \rangle : (x,X) \in \mathcal{F}^{R1}_\rho \right\} . \end{aligned}$$

Clearly, we have $\mathcal{F}^{R1}_\rho \subseteq \mathcal{F}^{R1}$ for $1\le \rho \le n$, where $\mathcal{F}^{R1}$ is given by (10). Our next result gives a description of $\mathcal{F}^{R1}_\rho$ in closed form for each $\rho \in \{1,\ldots ,n\}$.

#### Lemma 3.2

(i) For $\rho =1$, we have $\mathcal{F}^{R1}_1 = \left\{ (x,X) \in \mathbb {R}^n \times \mathcal{S}^n: e^\top x = 1, \quad X =\right. \left. {{\,\textrm{Diag}\,}}(x), \quad x \ge 0 \right\}$.
(ii) For each $\rho \in \{2,3,\ldots ,n\}$, we have $\mathcal{F}^{R1}_\rho = \mathcal{F}^{R1}$, where $\mathcal{F}^{R1}$ is given by (10).

#### Proof

(i) Let $$( x, X) \in \mathcal{F}^{R1}_1$$. Then $$e^\top x=1$$ and $$x\ge 0$$. Moreover, there exists $$( u, U, R) \in \mathbb {R}^n \times \mathcal{S}^n \times \mathbb {R}^{n \times n}$$ such that (*x*, *u*, *X*, *U*, *R*) is (R1($$\rho $$))-feasible with $$\rho =1$$. Since $$ U \ge 0$$ by Lemma 3.1, $${{\,\textrm{diag}\,}}( U) = u$$, and $$ U e = u$$, we obtain $$ U = {{\,\textrm{Diag}\,}}( u)$$. Since $$ R - U \le 0$$ and $$ R \ge 0$$ by Lemma 3.1, we obtain that *R* is a diagonal matrix. Similarly, using $$ X - R^\top \le 0$$, we conclude that *X* is a diagonal matrix. Since $$ X e = x$$, we obtain that $$ X = {{\,\textrm{Diag}\,}}( x)$$. Conversely, if $$e^\top x = 1$$, $$ X = {{\,\textrm{Diag}\,}}( x)$$, and $$ x \ge 0$$, then it is easy to verify that $$(x, u, X, U, R) = ( x, x, X, X, X)$$ is (R1($$\rho $$))-feasible. It follows that $$( x, X) \in \mathcal{F}^{R1}_1$$. (ii) Let $$\rho \in \{2,3,\ldots ,n\}$$. We clearly have $$\mathcal{F}^{R1}_\rho \subseteq \mathcal{F}^{R1}$$. Evidently, $$ \mathcal{F}^{R1}$$ is a polytope, so for the reverse inclusion, it suffices to show that each vertex of $$\mathcal{F}^{R1}$$ belongs to $$\mathcal{F}^{R1}_\rho $$. By Proposition 3.1, the set of vertices of $$\mathcal{F}^{R1}$$ is given by (11). If $$( x, X) = (e^i, e^i (e^i)^\top )$$ for some $$i = 1,\ldots ,n$$, then choose an arbitrary $$ u \in \{0,1\}^n$$ such that $$ u_i = 1$$ and $$e^\top u = \rho $$. If, on the other hand, $$( x, X) = (\frac{1}{2}(e^i + e^j), \frac{1}{2}(e^i (e^j)^\top +e^j (e^i)^\top ))$$ for some $$1 \le i < j \le n$$, then choose an arbitrary $$ u \in \{0,1\}^n$$ such that $$ u_i = 1$$, $$ u_j = 1$$, and $$e^\top u = \rho $$. In both cases then, define $$ R = x u^\top $$ and $$ U = u u^\top $$. It is easy to verify that $$( x, u, X, U, R) \in \mathbb {R}^n \times \mathbb {R}^n \times \mathcal{S}^n \times \mathcal{S}^n \times \mathbb {R}^{n \times n}$$ is (R1($$\rho $$))-feasible, which implies that each vertex of $$\mathcal{F}^{R1}$$ belongs to $$\mathcal{F}^{R1}_\rho $$. We conclude that $$\mathcal{F}^{R1}_\rho = \mathcal{F}^{R1}$$. $$\square $$

Lemma 3.2 immediately gives rise to the following results.

#### Corollary 3.1

(i) For $\rho = 1$, (R1($\rho$)) is exact, i.e., $\ell ^{R1}_1 (Q) = \ell _1(Q)$.
(ii) For each $\rho \in \{2,3,\ldots ,n\}$, we have $\ell ^{R1} (Q) = \ell ^{R1}_\rho (Q) = \min \limits _{1\le i \le j \le n} Q_{ij}$.

#### Proof

Both assertions follow from Lemma 3.2, Lemma 2.1, Proposition 3.1 and (15). $\square$

We arrive at the following exactness result for the classical RLT relaxation of sparse StQPs:

#### Theorem 3.1

(R1($\rho$)) is exact (i.e., $\ell ^{R1}_\rho (Q) = \ell _\rho (Q)$) if and only if $\rho = 1$ or $\min \limits _{1\le i \le j \le n} Q_{ij} = \min \limits _{1\le k \le n} Q_{kk}$.

#### Proof

By Corollary 3.1(i), (R1($\rho$)) is exact for $\rho = 1$. Let $\rho \in \{2,3,\ldots ,n\}$. If (R1($\rho$)) is exact, then Lemma 2.1 and Corollary 3.1(ii) imply that $\ell ^{R1}_\rho (Q) = \min \limits _{1\le i \le j \le n} Q_{ij} = \ell ^{R1} (Q) \le \ell (Q) \le \ell _\rho (Q) = \ell ^{R1}_\rho (Q) = \ell ^{R1} (Q)$. The claim follows from Proposition 3.1. Conversely, if $\min \limits _{1\le i \le j \le n} Q_{ij} = \min \limits _{1\le k \le n} Q_{kk}$, then $\ell ^{R1}_\rho (Q) = \ell ^{R1} (Q) = \min \limits _{1\le i \le j \le n} Q_{ij} = \min \limits _{1\le k \le n} Q_{kk} = \ell (Q) = \ell _\rho (Q)$ by Lemma 2.1, Corollary 3.1(ii), and Proposition 3.1. Therefore, (R1($\rho$)) is exact. $\square$

By Theorem 3.1, (R1($\rho$)) is exact if and only if $\rho = 1$ or (StQP) itself already has a 1-sparse optimal solution. Otherwise, in view of Lemma 2.1 and the relation $\ell ^{R1} (Q) \le \ell (Q)$, it follows from Corollary 3.1 that, for each $\rho \ge 2$, the lower bound $\ell ^{R1}_\rho (Q)$ arising from (R1($\rho$)) is, in general, quite weak as it already agrees with the lower bound $\ell ^{R1} (Q)$ obtained from the RLT relaxation (R1) of (StQP).

### SDP Relaxation

In this section, we consider the standard Shor relaxation of (StQP($\rho$)) in relation to that of (StQP).

The Shor relaxation of (StQP) is given by 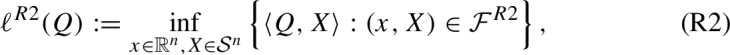 where the closed convex set

16 $$\begin{aligned} \mathcal{F}^{R2}:= \left\{ (x,X) \in \mathbb {R}^n \times \mathcal{S}^n: e^\top x = 1, \quad x \ge 0, \quad X \succeq x x^\top \right\} \, \end{aligned}$$

is not necessarily bounded, which necessitates the use of ‘$\inf$’ in (R2). Indeed, we have the following well-known result about (R2); we include a short proof for the sake of completeness.

#### Lemma 3.3

If $Q \succeq 0$, then (R2) is exact (i.e., $\ell ^{R2}(Q) = \ell (Q)$). If $Q \not \succeq 0$, then $\ell ^{R2}(Q) = -\infty$.

#### Proof

If $Q \succeq 0$, then, for any (R2)-feasible solution $( x, X) \in \mathbb {R}^n \times \mathcal{S}^n$, we have $\langle Q, X \rangle \ge x^\top Q x$ since $X \succeq x x^\top$, which implies that $\ell (Q) \ge \ell ^{R2}(Q) \ge \ell (Q)$. If $Q \not \succeq 0$, then there exists $d \in \mathbb {R}^n$ such that $d^\top Q d < 0$. Let $x \in \mathbb {R}^n$ be any feasible solution of (StQP) and let $X (\lambda ) = x x^\top + \lambda d d^\top$, where $\lambda \ge 0$. The assertion follows by observing that $( x, X (\lambda )) \in \mathcal{F}^{R2}$ for each $\lambda \ge 0$ and that the objective function of (R2) evaluated at $( x, X (\lambda ))$ tends to $-\infty$ as $\lambda \rightarrow \infty$. $\square$

The Shor relaxation of (StQP($\rho$)) is given by 
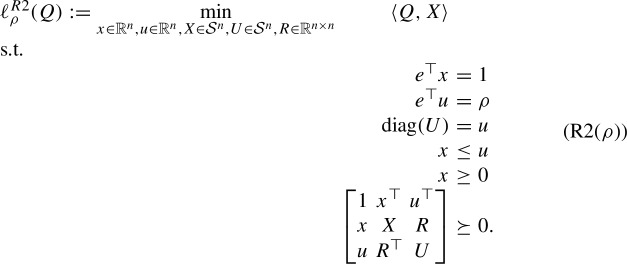
 Note that the constraint $u \le e$ *is* implied by ${{\,\textrm{diag}\,}}(U) = u$ and the semidefiniteness constraint.

Similarly to the RLT relaxation of (StQP($\rho$)), let us introduce the following projection of the feasible region of (R2($\rho$)) onto (*x*, *X*):

17 $$\begin{aligned} \mathcal{F}^{R2}_\rho:= &   \left\{ (x,X) \in \mathbb {R}^n \times \mathcal{S}^n: (x,u,X,U,R) \text { is}~R2(\rho )\text {-feasible for some }\right. \nonumber \\  &   \left. (u,U,R) \in \mathbb {R}^n \times \mathcal{S}^n \times \mathbb {R}^{n \times n}\right\} \,. \end{aligned}$$

We again observe that

18 $$\begin{aligned} \ell ^{R2}_\rho (Q) = \min \limits _{(x,X) \in \mathbb {R}^n \times \mathcal{S}^n}\left\{ \langle Q, X \rangle : (x,X) \in \mathcal{F}^{R2}_\rho \right\} . \end{aligned}$$

Our next result gives a complete description of $\mathcal{F}^{R2}_\rho$ for each $\rho = 1,2,\ldots ,n$.

#### Lemma 3.4

For each $\rho \in \{1,2,\ldots ,n\}$, we have $\mathcal{F}^{R2}_\rho = \mathcal{F}^{R2}$, where $\mathcal{F}^{R2}$ is given by (16).

#### Proof

We clearly have $\mathcal{F}^{R2}_\rho \subseteq \mathcal{F}^{R2}$. For the reverse inclusion, let $( x, X) \in \mathcal{F}^{R2}$ so that $e^\top x=1$ and $x\ge 0$, so also $x\le e$. Furthermore $X = x x^\top + M$ for some $M \succeq 0$. Define $u = x + \left( \frac{\rho - 1}{n-1}\right) (e - x)$ so that $e^\top u = \rho$ and $0 \le x \le u \le e$. Let $R = x u^\top$ and $U = u u^\top + D$, where $D \in \mathcal{S}^n$ is a diagonal matrix such that $D_{jj} = u_j - ( u_j)^2 \ge 0,~j = 1,\ldots ,n$. Note that ${{\,\textrm{diag}\,}}( U) = u$ and

$$\begin{aligned} \begin{bmatrix} X &  R \\ R^\top &  U \end{bmatrix} - \begin{bmatrix} x \\ u \end{bmatrix} \begin{bmatrix} x \\ u \end{bmatrix}^\top = \begin{bmatrix} M &  0 \\ 0 &  D \end{bmatrix} \succeq 0. \end{aligned}$$

By the Schur complement lemma, it follows that $( x, u, X, U, R) \in \mathbb {R}^n \times \mathbb {R}^n \times \mathcal{S}^n \times \mathcal{S}^n \times \mathbb {R}^{n\times n}$ is (R2($\rho$))-feasible. Therefore $( x, X) \in \mathcal{F}^{R2}_\rho$. $\square$

Lemma 3.4 reveals that none of the feasible solutions of $\mathcal{F}^{R2}$ is cut off in the projection of the feasible region of (R2($\rho$)) for any choice of $\rho \in \{1,2,\ldots ,n\}$. In view of (18), we obtain the following corollary.

#### Corollary 3.2

For any $\rho \in \{1,\ldots ,n\}$, we have $\ell ^{R2}_\rho (Q) = \ell ^{R2}(Q)$.

#### Proof

The assertion follows from (R2), (18), and Lemma 3.4. $\square$

Now we obtain the following exactness result for the Shor relaxation of the sparse StQP:

#### Theorem 3.2

(R2($\rho$)) is exact (i.e., $\ell ^{R2}_\rho (Q) = \ell _\rho (Q)$) if and only if $Q \succeq 0$ and (StQP) has a $\rho$-sparse optimal solution.

#### Proof

The assertion follows from Lemma 3.3, Corollary 3.2, and Lemma 2.1. $\square$

Theorem 3.2 shows that (R2($\rho$)) provides a finite lower bound if and only if $Q \succeq 0$. Furthermore, in this case, the bound is tight if and only if the problem (StQP) without any sparsity constraint already has a $\rho$-sparse optimal solution. It follows that (R2($\rho$)), in general, is a weak relaxation. We close this section by specializing Theorem 3.2 to the particular case with a rank-one $Q \in \mathcal{S}^n$.

#### Corollary 3.3

Let $Q = vv^\top$, where $v \in \mathbb {R}^n$. If $v \in \mathbb {R}^n_+$ or $-v \in \mathbb {R}^n_+$ or $v_i = 0$ for some $i \in \{1,\ldots ,n\}$, then $\ell ^{R2}_\rho (Q) = \ell _\rho (Q)$ for each $\rho \in \{1,\ldots ,n\}$. Otherwise, $\ell ^{R2}_1(Q) < \ell _1(Q)$ and $\ell ^{R2}_\rho (Q) = \ell _\rho (Q)$ for each $\rho \in \{ 2,\ldots ,n\}$.

#### Proof

Let $Q = vv^\top$, where $v \in \mathbb {R}^n$. Note that $x^\top Q x = (v^\top x)^2 \ge 0$ for each $x \in \mathbb {R}^n$. If $v \in \mathbb {R}^n_+$ (resp., $-v \in \mathbb {R}^n_+$ or $v_i = 0$ for some $i \in \{1,\ldots ,n\}$), then (StQP) has a 1-sparse optimal solution given by $e^j \in \mathbb {R}^n$, where $j = \arg \min \limits _{1\le i \le n} v_i$ (resp., $j = \arg \min \limits _{1\le i \le n}(-v_i)$ or $j = i$). The assertion follows from Theorem 3.2. Otherwise, there exist $i \in \{1,\ldots ,n\}$ and $j \in \{1,\ldots ,n\}$ such that $v_i< 0 < v_j$. Therefore, setting $x= \frac{v_j}{v_j-v_i}\, e^i - \frac{v_i}{v_j-v_i}\, e^j$, we obtain $x \in F$ and $x^\top Q x = (v^\top x)^2 = 0 = \ell (Q)$. On the other hand $\ell _1 (Q) = \min \limits _{1\le k \le n}Q_{kk} = \min \limits _{1\le k \le n}v_{k}^2 > 0$ by Lemma 2.1. $\square$

A comparison of Corollary 3.3 and Theorem 3.1 reveals that the Shor relaxation (R2($\rho$)) can be strictly weaker than the RLT relaxation (R1($\rho$)) for $\rho = 1$, even when $Q \succeq 0$.

## SDP-RLT Relaxation

In this section, we consider the SDP-RLT relaxations of (StQP($\rho$)) and (StQP) obtained by combining the corresponding RLT relaxations and SDP relaxations presented in Sect. 3.1 and Sect. 3.2, respectively. In particular, our objective is to shed light on the properties of the combined relaxation in relation to those of the two individual relaxations.

The SDP-RLT relaxation of (StQP) is given by 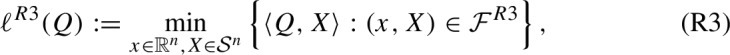 where

19 $$\begin{aligned} \mathcal{F}^{R3}:= \left\{ (x,X) \in \mathbb {R}^n \times \mathcal{S}^n: e^\top x = 1, \quad X e = x, \quad x \ge 0, \quad X \ge 0, \quad X \succeq x x^\top \right\} .\nonumber \\ \end{aligned}$$

The well-known doubly nonnegative (DNN) relaxation of (StQP) is given by 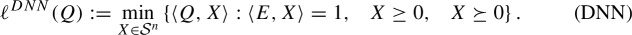 First, we establish the connection between the SDP-RLT relaxation (R3) and the DNN relaxation (DNN).

### Lemma 4.1

(*x*, *X*) is (R3)-feasible if and only if *X* is (DNN)-feasible.

### Proof

If (*x*, *X*) is (R3)-feasible, then $\langle E, X \rangle = e^\top X e = e^\top x = 1$, $X \ge 0$, and $X \succeq 0$ since $X \succeq x x^\top$ and $x x^\top \succeq 0$. Therefore, *X* is (DNN)-feasible.

Conversely, let *X* be (DNN)-feasible. Since $X \succeq 0$, there exists $A \in \mathbb {R}^{n \times n}$ such that $X = A A^\top$. Let $x = X e = A A^\top e = A w$, where $w = A^\top e$. Clearly, $x \ge 0$ since $X \ge 0$ and $e^\top x = e^\top X e = \langle E, X \rangle = \Vert w\Vert ^2_2 = 1$. Finally, $X - x x^\top = A A^\top - A w w^\top A^\top = A \left( I - w w^\top \right) A^\top \succeq 0$ since $I - w w^\top \succeq 0$ due to $\Vert w\Vert _2 = 1$. $\square$

By Lemma 4.1, the SDP-RLT relaxation (R3) and the DNN relaxation (DNN) are equivalent, i.e., $\ell ^{R3}(Q) = \ell ^{DNN}(Q)$. Therefore, a complete description of instances of (StQP) that admit exact SDP-RLT relaxations is given below.

### Theorem 4.1

(Gökmen and Yıldırım  [21]) (R3) is exact (i.e., $$\ell ^{R3}(Q) = \ell (Q)$$) if and only if (i) $$n \le 4$$; or (ii) $$n \ge 5$$ and there exist $$ x \in F$$, $$ P \succeq 0$$, $$N \in \mathcal{S}^n$$, $$ N \ge 0$$, $$ \lambda \in \mathbb {R}$$ such that $$ P x = 0$$, $$ x^\top N x = 0$$, and $$Q = P + N + \lambda E$$. Furthermore, for any such decomposition, $$ x \in F$$ is an optimal solution of (StQP) and $$\ell ^{R3}(Q) = \ell (Q) = \lambda $$.

We next consider the SDP-RLT relaxation of (StQP($\rho$)): 
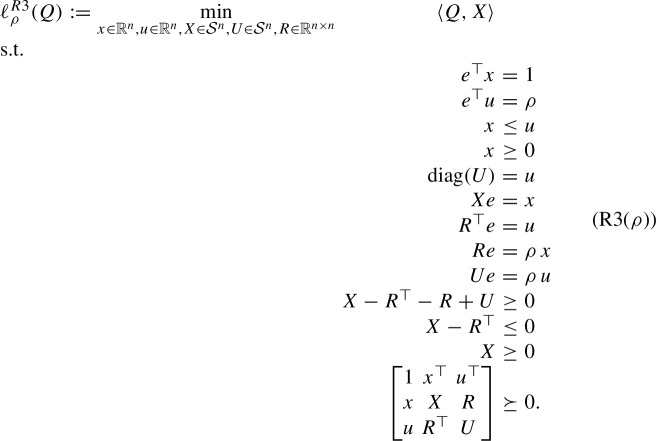


Similar to the RLT relaxation (R1($\rho$)), we remark that Lemma 3.1 also holds for (R3($\rho$)).

### A Simplification and Relation to Doubly Nonnegative Relaxation

In this section, we first present a simplified formulation of (R3($\rho$)). We then establish a similar relation between (R3($\rho$)) and a particular doubly nonnegative (DNN) relaxation of (StQP($\rho$)).

Consider the following problem:
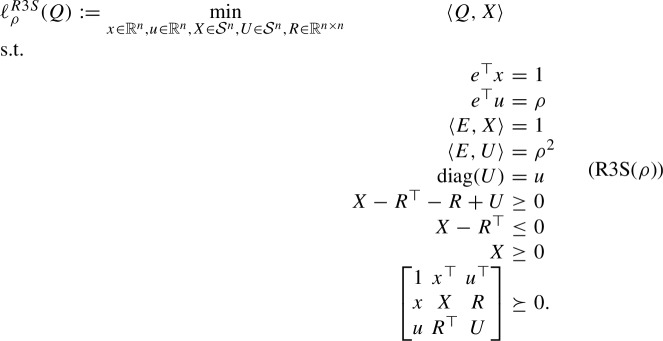
 Our next result establishes the equivalence between (R3($\rho$)) and (R3S($\rho$)).

#### Lemma 4.2

(*x*, *u*, *X*, *U*, *R*) is (R3($\rho$))-feasible if and only if it is (R3S($\rho$))-feasible.

#### Proof

Let (*x*, *u*, *X*, *U*, *R*) be (R3($\rho$))-feasible. Then, $\langle E, X \rangle = e^\top X e = e^\top x = 1$ and $\langle E, U \rangle = e^\top U e = \rho \, e^\top u = \rho ^2$, which implies that (*x*, *u*, *X*, *U*, *R*) is (R3S($\rho$))-feasible.

Conversely, suppose that (*x*, *u*, *X*, *U*, *R*) is (R3S($\rho$))-feasible. Using the Schur complement lemma,

$$\begin{aligned} {\begin{bmatrix} X &  R \\ R^\top &  U \end{bmatrix} = \begin{bmatrix} x \\ u\end{bmatrix} \begin{bmatrix} x \\ u\end{bmatrix} ^\top + \sum \limits _{k \in K} \begin{bmatrix} a^k \\ b^k \end{bmatrix} \begin{bmatrix} a^k \\ b^k \end{bmatrix} ^\top , } \end{aligned}$$

where *K* is a finite set and ${a}^k \in \mathbb {R}^n$ and ${b}^k \in \mathbb {R}^n$ for each $k \in K$. Combining this decomposition with $e^\top x = 1$, $e^\top u = \rho$, $\langle E, X \rangle = 1$, and $\langle E, U \rangle = \rho ^2$, we obtain

$$\begin{aligned} e^\top a^k = e^\top b^k = 0, \quad k \in K, \end{aligned}$$

which, in turn, implies that $X e = x$, $R^\top e = u$, $R e = \rho \,x$, and $U e = \rho \, u$. It follows from Lemma 3.1 that (*x*, *u*, *X*, *U*, *R*) is (R3($\rho$))-feasible. $\square$

In comparison with (R3($\rho$)), we remark that (R3S($\rho$)) has $4n - 2$ fewer linear equality constraints, which may translate into significant computational advantages.

We next discuss the relation between (R3S($\rho$)) and the DNN relaxation of (StQP($\rho$)). After introducing slack variables $y = u - x \ge 0$, according to [13], we obtain the following copositive relaxation of (StQP($\rho$)): 
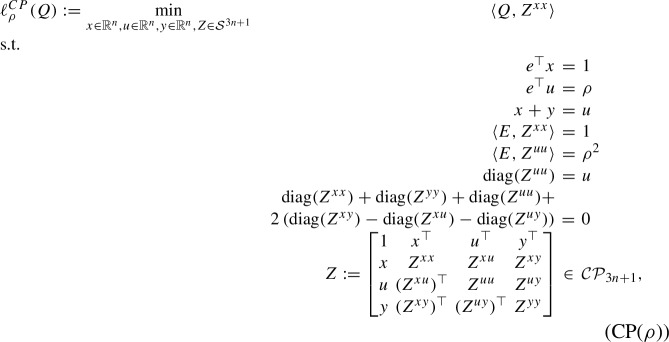


where $\mathcal{C}\mathcal{P}_n= \left\{ M \in \mathcal{S}^n: M = A A^\top \quad \text {for some } A \in \mathbb {R}^{n \times k} \text { such that } A \ge 0 \right\}$ denotes the convex cone of completely positive matrices. We remark that (CP($\rho$)) is not necessarily an exact relaxation of (StQP($\rho$)) since Burer’s key condition [13] is not satisfied without explicitly adding the constraints $u \le e$ to (StQP($\rho$)).

The DNN relaxation of (CP($\rho$)) is given by replacing the computationally intractable conic constraint $Z \in \mathcal{C}\mathcal{P}_{3n+1}$ by the tractable outer approximation given by the cone of doubly nonnegative matrices, i.e., $Z \ge 0$ and $Z \succeq 0$. Let us denote the resulting DNN relaxation by (DNN($\rho$)). We next establish the equivalence between (R3S($\rho$)) and (DNN($\rho$)).

#### Theorem 4.2

(*x*, *u*, *X*, *U*, *R*) is (R3S($\rho$))-feasible if and only if (*x*, *u*, *y*, *Z*) is (DNN($\rho$))-feasible, where $y \in \mathbb {R}^n$ and $Z \in \mathcal{S}^{3n + 1}$ are given by

20 $$\begin{aligned} Z = \begin{bmatrix} 1 &  x^\top &  u^\top &  y^\top \\ x&  Z^{xx}& Z^{xu} &  Z^{xy}\\ u& (Z^{xu})^\top & Z^{uu} &  Z^{uy}\\ y&  (Z^{xy})^\top & (Z^{uy})^\top & Z^{yy}\\ \end{bmatrix} = \begin{bmatrix} 1 &  \quad x^\top &  \quad u^\top &  \quad u^\top - x^\top \\ x &  \quad X &  \quad R &  \quad R - X \\ u &  \quad R^\top &  \quad U &  \quad U - R^\top \\ u - x &  \quad R^\top - X &  \quad U - R &  \quad U - R- R^\top + X\\ \end{bmatrix}.\nonumber \\ \end{aligned}$$

#### Proof

Let (*x*, *u*, *X*, *U*, *R*) be (R3S($\rho$))-feasible and let *Z* be given by (20). By Lemma 3.1, $Z \ge 0$. Furthermore,

$$\begin{aligned} Z = \begin{bmatrix}1 &  x^\top &  u^\top &  y^\top \\ x&  Z^{xx}& Z^{xu} &  Z^{xy}\\ u& (Z^{xu})^\top & Z^{uu} &  Z^{uy}\\ y&  (Z^{xy})^\top & (Z^{uy})^\top & Z^{yy}\\ \end{bmatrix} = \begin{bmatrix} 1 &  0^\top &  0^\top \\ 0 &  I &  0\\ 0 &  0 &  I \\ 0 &  -I & I \\ \end{bmatrix} \begin{bmatrix} 1 &  x^\top &  u^\top \\ x&  X& R \\ u& R^\top & U \end{bmatrix} \begin{bmatrix} 1 &  0^\top &  0^\top \\ 0 &  I &  0\\ 0 &  0 &  I \\ 0 &  -I & I \\ \end{bmatrix} ^\top , \end{aligned}$$

which implies that $Z \succeq 0$. We only need to verify the last equality constraint of (DNN($\rho$)). By (20),

$$\begin{aligned}  &   {{\,\textrm{diag}\,}}(X) + {{\,\textrm{diag}\,}}(U - R- R^\top + X) + {{\,\textrm{diag}\,}}(U) \\  &   \qquad + 2 \left( {{\,\textrm{diag}\,}}(R - X) - {{\,\textrm{diag}\,}}(R) - {{\,\textrm{diag}\,}}(U - R^\top ) \right) = 0, \end{aligned}$$

which implies that (*x*, *u*, *y*, *Z*) is (DNN($\rho$))-feasible.

Conversely, let (*x*, *u*, *y*, *Z*) be (DNN($\rho$))-feasible. Let us define $(X, U, R) = (Z^{xx}, Z^{uu}, Z^{xu})$. By (20),

$$\begin{aligned} \begin{bmatrix} 1 &  x^\top &  u^\top \\ x &  X &  R \\ u &  R^\top &  U \end{bmatrix} = \begin{bmatrix}1 &  x^\top &  u^\top \\ x&  Z^{xx}& Z^{xu} \\ u& (Z^{xu})^\top & Z^{uu} \end{bmatrix} \succeq 0, \end{aligned}$$

and $X - R^\top - R + U = Z^{yy} \ge 0$, $R^\top - X = (Z^{xy})^\top \ge 0$, and $X = Z^{xx} \ge 0$. Therefore, (*x*, *u*, *X*, *U*, *R*) is (R3S($\rho$))-feasible. This completes the proof. $\square$

By Theorem 4.2, we conclude that the SDP-RLT relaxation (R3S($\rho$)) is an equivalent reformulation of the DNN relaxation (DNN($\rho$)) of (CP($\rho$)) with a simpler conic constraint in a smaller dimension. We refer the reader to [9] for significant computational savings from similar reductions of other DNN relaxations of the sparse StQP.

#### Remark 4.1

By adding the redundant constraint $u \le e$ to (StQP($\rho$)) and introducing another set of slack variables $v = e - u \ge 0$, the copositive relaxation of (StQP($\rho$)), denoted by (CP$^\prime$($\rho ))$, is exact since Burer’s key condition is now satisfied [13]. Similar to (DNN($\rho$)), we obtain the DNN relaxation of (CP$^\prime$($\rho ))$, denoted by (DNN$^\prime$($\rho$)), by replacing the completely positive cone with the DNN cone. An interesting question is whether the two DNN relaxations (DNN($\rho$)) and (DNN$^\prime$($\rho$)) are, in fact, equivalent. To that end, consider an instance of (StQP($\rho$)), where

$$\begin{aligned} Q = \begin{bmatrix} 1 &  -1 &  1 &  1 &  -1 \\ -1 &  1 &  -1 &  1 &  1 \\ 1 &  -1 &  1 &  -1 &  1 \\ 1 &  1 &  -1 &  1 &  -1 \\ -1 &  1 &  1 &  -1 &  1 \end{bmatrix} \end{aligned}$$

is the well-known Horn matrix. By varying $\rho \in \{1,\ldots ,5\}$, the exact values denoted by $\ell _{\rho }(Q)$ and the lower bounds obtained from the two DNN relaxations are presented in Table 1.

**Table 1:** Lower bounds from (DNN($\rho$)) and (DNN$^\prime$($\rho ))$

$\rho$	$\ell _{\rho }(Q)$	(DNN($\rho$))	(DNN$^\prime$($\rho$))
1	1	1	1
2	0	$-$**0.1056**	**0**
3	0	$-$0.1056	$-$0.1056
4	0	$-$0.1056	$-$0.1056
5	0	$-$0.1056	$-$0.1056

Bold indicates the case of difference

As illustrated by Table 1 for $\rho = 2$, (DNN($\rho$)) can be strictly weaker than (DNN$^\prime$($\rho$)). Therefore, the two DNN relaxations are not equivalent in general. However, we remark that the simplified formulation (R3S($\rho$)) of (DNN($\rho$)) provides a good trade-off between the computation time and the quality of the lower bound. The reader is referred to [9] for extensive computational results on the quality of the bounds arising from (DNN$^\prime$($\rho$)).

### Projected Feasible Sets and Their Inner Approximations

Similar to the RLT and SDP relaxations, consider the projection of the feasible region of (R3($\rho$)) (or, equivalently of (R3S($\rho$))) onto (*x*, *X*) given by

21 $$\begin{aligned} \mathcal{F}^{R3}_\rho= &   \left\{ (x,X) \in \mathbb {R}^n \times \mathcal{S}^n: (x,u,X,U,R) \text { is } (R3(\rho ))\text { -feasible for some }\right. \nonumber \\  &   \left. (u,U,R) \in \mathbb {R}^n \times \mathcal{S}^n \times \mathbb {R}^{n \times n} \right\} . \end{aligned}$$

In a similar manner as above, we have

22 $$\begin{aligned} \ell ^{R3}_\rho (Q) = \min \limits _{(x,X) \in \mathbb {R}^n \times \mathcal{S}^n}\left\{ \langle Q, X \rangle : (x,X) \in \mathcal{F}^{R3}_\rho \right\} \,. \end{aligned}$$

It is also easy to see that

23 $$\begin{aligned} \mathcal{F}^{R3}_\rho \subseteq \mathcal{F}^{R1}_\rho \cap \mathcal{F}^{R2}_\rho \,, \end{aligned}$$

where $\mathcal{F}^{R1}_\rho$ and $\mathcal{F}^{R2}_\rho$ are given by (14) and (17), respectively. Therefore,

24 $$\begin{aligned} \max \{\ell ^{R1}_\rho (Q), \ell ^{R2}_\rho (Q)\} \le \ell ^{R3}_\rho (Q) \le \ell _\rho (Q) \quad \text {for all }\rho \in \{ 1,\ldots ,n\}\,, \end{aligned}$$

which implies that (R3($\rho$)) is at least as tight as each of (R1($\rho$)) and (R2($\rho$)).

Our first result follows from the previous results on weaker relaxations.

#### Corollary 4.1

If (i) $\rho = 1$, or (ii) $\min \limits _{1\le i \le j \le n} Q_{ij} = \min \limits _{1\le k \le n} Q_{kk}$, or (iii) $Q \succeq 0$ and (StQP) has a $\rho$-sparse optimal solution, then (R3($\rho$)) is exact.

#### Proof

The assertion follows from Theorem 3.1, Theorem 3.2, and (24). $\square$

We now focus on the sets $\mathcal{F}^{R3}_\rho$, $\rho \in \{ 1,\ldots ,n\}$. By Lemma 3.2, Lemma 3.4, (19), and (23),

25 $$\begin{aligned} \mathcal{F}^{R3}_1\subseteq &   \left\{ (x,X) \in \mathbb {R}^n \times \mathcal{S}^n: e^\top x = 1, \, X = {{\,\textrm{Diag}\,}}(x), \, X \succeq x x^\top , \, x \ge 0\right\} \subseteq \mathcal{F}^{R3}, \nonumber \\ \end{aligned}$$

26 $$\begin{aligned} \mathcal{F}^{R3}_\rho\subseteq &   \left\{ (x,X) \in \mathbb {R}^n \times \mathcal{S}^n: e^\top x = 1, \, Xe = x, \, X \succeq x x^\top , \, X \ge 0, \,x \ge 0\right\} = \mathcal{F}^{R3}, \;\rho \ge 2. \nonumber \\ \end{aligned}$$

Next, we consider inner approximations of the sets $\mathcal{F}^{R3}_\rho$, where $\rho \in \{1,2,\dots ,n\}$.

#### Lemma 4.3

For any fixed $\rho \in \{ 1,\ldots ,n\}$, consider the corresponding formulation (StQP($\rho$)). Then, we have

27 $$\begin{aligned} {{\,\textrm{conv}\,}}\left( \left\{ (x,x x^\top ): x \in F_\rho \right\} \right) \subseteq \mathcal{F}^{R3}_\rho \,, \end{aligned}$$

where $F_\rho$ and $\mathcal{F}^{R3}_\rho$ are given by (5) and (21), respectively.

#### Proof

For any (StQP($\rho$))-feasible solution $( x, u) \in \mathbb {R}^n \times \mathbb {R}^n$, we define $X = x x^\top$, $R = x u^\top$, and $U = u u^\top$. Then obviously $( x, u, X, U, R) \in \mathbb {R}^n \times \mathbb {R}^n \times \mathcal{S}^n \times \mathcal{S}^n \times \mathbb {R}^{n \times n}$ is (R3($\rho$))-feasible. The claim now follows by (21) and the convexity of $\mathcal{F}^{R3}_\rho$. $\square$

In the remainder of this section, we identify further properties of the sets $\mathcal{F}^{R3}_\rho$, where $\rho \in \{1,2,\dots ,n\}$ and their implications on the tightness of the lower bound $\ell ^{R3}_\rho (Q)$.

### The Extremely Sparse Case $\rho =1$

In this section, we give an exact description of the set $\mathcal{F}^{R3}_1$ and discuss its implications. We start with a technical lemma.

#### Lemma 4.4

Let $a \in \mathbb {R}^n$ and define $A:={{\,\textrm{Diag}\,}}(a) - aa^\top$. Then, the following statements are equivalent:

- $$a \in \mathbb {R}^n_+$$ and $$e^\top a \le 1$$;
- *A* is positive-semidefinite.

#### Proof

(a) implies (b) by diagonal dominance: if $a \in \mathbb {R}^n_+$ and $e^\top a \le 1$, then ${{\,\textrm{Diag}\,}}(a) - aa^\top$ is diagonally dominant since

$$\begin{aligned}  &   a_i - a_i^2 - \sum \limits _{j \in \{1,\ldots ,n\} \backslash \{i\}} \left| - a_i a_j \right| = a_i - a_i^2 - a_i \sum \limits _{j \in \{1,\ldots ,n\} \backslash \{i\}} a_j \\  &   \qquad \qquad = a_i \left( 1 - e^\top a \right) \ge 0, \quad i = 1,\ldots ,n\,. \end{aligned}$$

Hence *A* must be positive-semidefinite. It remains to show that (b) implies (a). Now, if *A* is positive-semidefinite, then its diagonal entries satisfy $a_i - a_i^2 \ge 0$, $i = 1,\ldots ,n$, which implies that $0 \le a \le e$. Furthermore, $e^\top \left( {{\,\textrm{Diag}\,}}(a) - aa^\top \right) e = e^\top a - (e^\top a)^2 \ge 0$, which implies that $e^\top a \le 1$. $\square$

By Lemma 4.4, it is easy to see that the constraint $X - x x^\top \succeq 0$ on the right-hand side of (25) is redundant. Therefore, by Lemma 3.2, we obtain

28 $$\begin{aligned} \mathcal{F}^{R3}_1 \subseteq \left\{ (x,X) \in \mathbb {R}^n \times \mathcal{S}^n: e^\top x = 1, \quad X = {{\,\textrm{Diag}\,}}(x), \quad x \ge 0\right\} = \mathcal{F}^{R1}_1. \end{aligned}$$

Our next result shows that the inclusion in (28) actually holds with equality, thereby yielding an exact description of $\mathcal{F}^{R3}_1$.

#### Lemma 4.5

We have

29 $$\begin{aligned} \mathcal{F}^{R3}_1= &   \mathcal{F}^{R1}_1 = {{\,\textrm{conv}\,}}\left( \left\{ (x,x x^\top ): x \in F_1 \right\} \right) \nonumber \\= &   {{\,\textrm{conv}\,}}\left( \left\{ (e^j,e^j (e^j)^\top ): j \in \{ 1,\ldots ,n \}\right\} \right) , \end{aligned}$$

where $\mathcal{F}^{R3}_1$ and $\mathcal{F}^{R1}_1$ are defined as in (21) and (14), respectively.

#### Proof

The assertion follows from the observation that $\mathcal{F}_1^{R1} = {{\,\textrm{conv}\,}}\left( \left\{ (e^j,e^j (e^j)^\top ): \right. \right. \left. \left. 1\le j \le n \right\} \right)$ in conjunction with Lemma 4.3 and (28). $\square$

Lemma 4.5 reveals that the SDP-RLT relaxation (R3($\rho$)) is identical to the RLT relaxation (R1($\rho$)) for $\rho = 1$: the semidefiniteness constraint in (R3($\rho$)) is redundant. A similar observation holds for (R3S(1)): arguing very much like in Lemma 3.2, one can show that the semidefiniteness constraint there is implied by the (additional) linear constraint $Ue=u$ borrowed from (R1(1)), arriving at $x=u$ and $X=U=R={{\,\textrm{Diag}\,}}(x)$, arguing with the help of Lemma 4.4.

### Case of Larger Sparsity $\rho \ge 2$

In this section, we focus on the sets $\mathcal{F}^{R3}_\rho$, where $\rho \in \{2,3,\ldots ,n\}$, and establish several properties and relations. Our first result strengthens the inner approximation of $\mathcal{F}^{R3}_\rho$ given by Lemma 4.3.

#### Lemma 4.6

We have

$$\begin{aligned} \left\{ (x,X) \in \mathcal{F}^{R3}: x \in F_\rho \right\} \subseteq \mathcal{F}^{R3}_\rho , \quad \text {{for} all }\rho \in \{ 2,\ldots ,n\}\,, \end{aligned}$$

where $F_\rho$, $\mathcal{F}^{R3}$, and $\mathcal{F}^{R3}_\rho$ are given by (5), (19) and (21), respectively.

#### Proof

Fix $\rho \in \{2,\ldots ,n\}$ and let $( x, X) \in \mathcal{F}^{R3}$ with $\Vert x \Vert _0 \le \rho$. Choose $u \in \{0,1\}^n$ such that $x \le u$ and $e^\top u = \rho$. Define $R = x u^\top$ and $U = u u^\top$. We claim that (*x*, *u*, *X*, *U*, *R*) is (R3S($\rho$))-feasible. Clearly, $\langle E, X \rangle = e^\top X e = e^\top x = 1$, $\langle E, U \rangle = (e^\top u)^2 = \rho ^2$, ${{\,\textrm{diag}\,}}( U) = u$, and $X \ge 0$. Since $X = x x^\top + M$ for some $M \succeq 0$, we obtain

$$\begin{aligned} \begin{bmatrix} X &  R \\ R^\top &  U \end{bmatrix} - \begin{bmatrix} x \\ u \end{bmatrix} \begin{bmatrix} x \\ u \end{bmatrix}^\top = \begin{bmatrix} M &  0 \\ 0 &  0 \end{bmatrix} \succeq 0. \end{aligned}$$

Next, we consider the constraint $$ X - R^\top \le 0$$. Since $$ X \ge 0$$ and $$ X e = x$$, we obtain $$0 \le X_{ij} \le \min \{ x_i, x_j\}$$ for each $$1 \le i \le j \le n$$. Therefore, if $$\min \{ x_i, x_j\} = 0$$, then $$ X_{ij} - u_i x_j = - u_i x_j \le 0$$. On the other hand, if $$\min \{ x_i, x_j\} > 0$$, then $$ u_i = 1$$, which implies that $$ X_{ij} - u_i x_j = X_{ij} - x_j \le 0$$. It follows that $$ X - R^\top \le 0$$.

Finally, we need to show that $$ X - R - R^\top + U \ge 0$$. For each $$1 \le i \le j \le n$$, if $$\min \{ x_i, x_j\} = 0$$, then $$ X_{ij} = 0$$ and $$\min \{ R_{ij}, R_{ji}\} = \min \{ x_i u_j, x_j u_i\} = 0$$. Therefore,

$$\begin{aligned} X_{ij} - R_{ij} - R_{ji} + U_{ij}= &   0-\max \{ R_{ij}, R_{ji}\}-0 + u_iu_j \\= &   - \max \{ x_i u_j, x_j u_i\} + u_i u_j \ge 0, \end{aligned}$$

since $$ x \le u$$. Here, we used the lattice identity $$v+w=\min \{ v,w\} + \max \{v,w\}$$. On the other hand, if $$\min \{ x_i, x_j\} > 0$$, then $$ u_i = u_j = 1$$, which implies that $$ X_{ij} - R_{ij} - R_{ji} + U_{ij} = X_{ij} - x_i - x_j + 1$$. For any $$1 \le i < j \le n$$, since $$ x_i + x_j \le 1$$, we clearly have $$ X_{ij} - x_i - x_j + 1 \ge 0$$ since $$ X \ge 0$$. Finally, if $$i = j$$, since $$ X \succeq 0$$ and $$ X e = x$$, we obtain

$$\begin{aligned} X_{ii} - 2 x_i + 1 = ( e^i - e)^\top X ( e^i - e) \ge 0, \quad i = 1,\ldots ,n, \end{aligned}$$

which completes the proof. $\square$

By Lemma 4.6, none of the solutions in $(x,X) \in \mathcal{F}^{R3}$ with $x \in F_\rho$ is cut off by the projection $\mathcal{F}^{R3}_\rho$. This observation gives rise to the following corollary.

#### Corollary 4.2

(i) For each $\rho \in \{2,\ldots ,n\}$, if there exists an optimal solution $( x, X) \in \mathbb {R}^n \times \mathcal{S}^n$ of (R3) such that $\Vert x \Vert _0 \le \rho$, then $\ell ^{R3}(Q) = \ell ^{R3}_\rho (Q)$.
(ii) We have $\mathcal{F}^{R3}_n = \mathcal{F}^{R3}$ and $\ell ^{R3}(Q) = \ell ^{R3}_n(Q)$.

#### Proof

(i) We clearly have $\ell ^{R3}(Q) \le \ell ^{R3}_\rho (Q)$ by (R3), (22), and (26). The reverse inequality follows from Lemma 4.6.
(ii) As $F_n=F$, the first equality follows from (26) and Lemma 4.6, and the second one from the first assertion (i). $\square$

By Corollary 4.2, we can identify a particular set of instances of (StQP($\rho$)) that admit an exact SDP-RLT relaxation.

#### Corollary 4.3

Let $$\rho \in \{2,\ldots ,n\}$$, $$ x \in F_\rho $$, and $$ \lambda \in \mathbb {R}$$. Let $$ P \succeq 0$$ be such that $$ P x = 0$$, and let $$N \in \mathcal{S}^n$$ be such that $$ N \ge 0$$ and $$ x^\top N x = 0$$. If $$Q = P + N + \lambda E$$, then the SDP-RLT relaxation (R3($$\rho $$)) is exact, i.e., $$\ell ^{R3}_\rho (Q) = \ell _\rho (Q)$$.

#### Proof

Under the hypotheses, Theorem 4.1 implies that $x \in F$ is an optimal solution of (StQP) and $\ell ^{R3}(Q) = \ell (Q) = \lambda$. The assertion follows from Corollary 4.2(i) and Lemma 2.1. $\square$

### Rank-One Elements of $\mathcal{F}^{R3}_\rho$

Recall that each solution $(x,X) \in \mathcal{F}^{R3}$, where $x \in F_\rho$, is retained in the projection $\mathcal{F}^{R3}_\rho ,~\rho = 1,\ldots ,n$ by Lemma 4.6. In this section, our goal is to shed light on the relations between $\mathcal{F}^{R3}_\rho$ and the set of solutions $(x,X) \in \mathcal{F}^{R3}$, where $\Vert x\Vert _0 > \rho$.

First, it follows from Lemma 4.3 and Lemma 4.5 that

30 $$\begin{aligned} \mathcal{F}^{R3}_1 \subseteq \mathcal{F}^{R3}_\rho \quad \text{ for } \text{ all } \rho \in \{ 2,\ldots ,n\}\,, \end{aligned}$$

which, in turn, implies that $(x,X) = (\frac{1}{n} e, \frac{1}{n} I) \in \mathcal{F}^{R3}_\rho$ for each $\rho \in \{1,\ldots ,n\}$ by Lemma 4.5. Therefore, for each $\rho \in \{1,\ldots ,{n-1}\}$, there exists $(x,X) \in \mathcal{F}^{R3}_\rho$ such that $\Vert x\Vert _0 > \rho$.

Let us restrict our attention to the subset of “rank-one solutions” $(x,X) \in \mathcal{F}^{R3}$, i.e., those with $\Vert x\Vert _0 = \nu > \rho$ and $X = x x^\top$. Note that $\langle Q, X \rangle = x^\top Q x$ for each rank-one solution. This, in turn, enables us to compare $\ell ^{R3}_\rho (Q)$ and $\ell _{\nu }(Q)$ for some $\nu > \rho$.

We start with the following result for $\rho = 1$.

#### Corollary 4.4

$( x, x x^\top ) \in \mathcal{F}^{R3}_1$ if and only if $x \in F_1$.

#### Proof

The claim follows from Lemma 4.5. $\square$

By Corollary 4.4, each rank-one solution $( x, x x^\top ) \in \mathcal{F}^{R3}$, where $\Vert x \Vert _0 > 1$, is cut off by $\mathcal{F}^{R3}_1$. We next focus on $\mathcal{F}^{R3}_\rho$ for $\rho \ge 2$. To that end, we first state a technical result about the feasible region of (R3($\rho$)).

#### Lemma 4.7

Let $\rho \in \{1,\ldots ,n\}$ and let $( x, u, X, U, R) \in \mathbb {R}^n \times \mathbb {R}^n \times \mathcal{S}^n \times \mathcal{S}^n \times \mathbb {R}^{n\times n}$ be (R3($\rho$))-feasible. Then,

31 $$\begin{aligned} \left( \rho - 2\right) u_i + 2 R_{ii} + (1 - \rho ) x_i - X_{ii} \ge 0, \quad \text{ for } \text{ all } i \in \{1,\ldots ,n\}\,. \end{aligned}$$

#### Proof

Suppose that $( x, u, X, U, R) \in \mathbb {R}^n \times \mathbb {R}^n \times \mathcal{S}^n \times \mathcal{S}^n \times \mathbb {R}^{n\times n}$ is (R3($\rho$))-feasible. Let us fix $i \in \{1,\ldots ,n\}$. For each $j \in \{1,\ldots ,n\}$ such that $j \ne i$, we have

$$\begin{aligned} U_{ij} - R_{ij} - R_{ji} + X_{ij} \ge 0. \end{aligned}$$

Therefore,

$$\begin{aligned} 0\le &   \sum \limits _{j \in \{1,\ldots ,n\} \backslash \{i\}} \left( U_{ij} - R_{ij} - R_{ji} + X_{ij}\right) \\= &   \left( \rho u_i - u_i \right) - \left( \rho x_i - R_{ii} \right) - \left( u_i - R_{ii}\right) + \left( x_i - X_{ii}\right) \\= &   \left( \rho - 2\right) u_i + 2 R_{ii} + (1 - \rho ) x_i - X_{ii}, \end{aligned}$$

where we used $${{\,\textrm{diag}\,}}( U) = u$$, $$ X e = x$$, $$ R^\top e = u$$, $$ R e = \rho \, x$$, and $$ U e = \rho \, u$$ in the second line. The assertion follows. $$\square $$

Using this technical result, we can establish the following result about rank-one solutions for $\rho = 2$.

#### Corollary 4.5

For each $x \in F$ such that $\Vert x\Vert _0 \ge 4$, we have $( x, x x^\top ) \not \in \mathcal{F}^{R3}_2$.

#### Proof

We prove the contrapositive. Let $\rho = 2$ and let $( x, x x^\top ) \in \mathcal{F}^{R3}_\rho$. Then, there exists $( u, U, R) \in \mathbb {R}^n \times \mathcal{S}^n \times \mathbb {R}^{n \times n}$ such that $( x, u, X, U, R) \in \mathbb {R}^n \times \mathbb {R}^n \times \mathcal{S}^n \times \mathcal{S}^n \times \mathbb {R}^{n\times n}$ is (R3($\rho$))-feasible, where $X = x x^\top$. Since $X = x x^\top$, it follows from the positive-semidefiniteness constraint and the Schur complement lemma that $R = x u^\top$. By Lemma 4.7, we obtain

$$\begin{aligned} \left( \rho - 2\right) u_i + 2 x_i u_i + (1 - \rho ) x_i - x_i^2 \ge 0 \quad \text{ for } \text{ all } i \in \{ 1,\ldots ,n\}\,. \end{aligned}$$

Using $$\rho = 2$$, for each $$i \in \{1,\ldots ,n\}$$ such that $$ x_i > 0$$, we obtain

$$\begin{aligned} u_i \ge \frac{1 + x_i}{2}\,. \end{aligned}$$

Summing over all $$i \in \{1,\ldots ,n\}$$ with $$ x_i > 0$$, and observing $$\sum \limits _{i:x_i>0} x_i= e^\top x= 1$$, we arrive at

$$\begin{aligned} 2 = \sum _i u_i \ge \sum _{i: x_i>0} u_i \ge \frac{\Vert x\Vert _0 + 1}{2}\,, \end{aligned}$$

which implies that $\Vert x \Vert _0 \le 3$. The assertion follows. $\square$

By Corollary 4.5, each rank-one solution $( x, x x^\top ) \in \mathcal{F}^{R3}$, where $\Vert x \Vert _0 > 3$, is cut off by $\mathcal{F}^{R3}_2$. Furthermore, for each $x \in F$ such that $\Vert x\Vert _0 = 3$, if $( x, x x^\top ) \in \mathcal{F}^{R3}$, then the proof of Corollary 4.5 implies that there exists a unique $u \in \mathbb {R}^n$ given by $u = \frac{1}{2}( x + e) = x + \frac{1}{2}(e - x)$ such that $( x, u, x x^\top , U, R) \in \mathbb {R}^n \times \mathbb {R}^n \times \mathcal{S}^n \times \mathcal{S}^n \times \mathbb {R}^{n\times n}$ is (R3($\rho$))-feasible. Our next result establishes that such a (R3($\rho$))-feasible solution can always be constructed and that the choice of *u* can be generalized to larger values of $\rho$.

#### Theorem 4.3

We have

32 $$\begin{aligned} \left\{ (x, x x^\top ) \in \mathcal{F}^{R3}: x \in F_{2 \rho - 1}\right\}\subseteq &   \mathcal{F}^{R3}_\rho \quad \text{ for } \text{ all } \rho \in \left\{ 2,\ldots ,\left\lfloor \textstyle {\frac{n+1}{2}} \right\rfloor \right\} , \end{aligned}$$

33 $$\begin{aligned} \left\{ (x, x x^\top ) \in \mathcal{F}^{R3}: x \in F\right\}\subseteq &   \mathcal{F}^{R3}_\rho \quad \text{ for } \text{ all } \rho \in \left\{ \left\lfloor \textstyle {\frac{n+1}{2}} \right\rfloor + 1, \ldots ,n\right\} , \end{aligned}$$

34 $$\begin{aligned} \left\{ (x, x x^\top ) \in \mathcal{F}^{R3}: x \in G_\rho \right\}\subseteq &   \mathcal{F}^{R3}_\rho \quad \text{ for } \text{ all } \rho \in \left\{ 2, \ldots , \left\lfloor \textstyle {\frac{n}{2}} \right\rfloor \right\} , \end{aligned}$$

where we define, for $\rho \in \left\{ 2, \ldots , \left\lfloor \textstyle {\frac{n}{2}} \right\rfloor \right\}$,

35 $$\begin{aligned} G_\rho:= &   \left\{ x \in F: \Vert x\Vert _0> 2 \rho - 1, \quad \max \limits _{1 \le i < j \le n: x_i x_j > 0} \frac{x_i x_j}{1 - x_i - x_j} \right. \nonumber \\  &   \left. \le \frac{(\rho - 1)(\rho - 2)}{\left( \Vert x\Vert _0 - 2\right) \left( \Vert x\Vert _0 - 2 \rho + 1\right) }\right\} \,. \end{aligned}$$

#### Proof

By Corollary 4.2(ii), we have $\mathcal{F}^{R3}_n = \mathcal{F}^{R3}$, which implies (33) for $\rho = n$. Therefore, let $\rho \in \{2,\ldots ,n-1\}$. By Lemma 4.6, it suffices to focus on rank-one solutions $(x, x x^\top )$, where $x\in F$ with $\Vert x\Vert _0 \ge \rho + 1$. We abbreviate $\nu :=\Vert x\Vert _0$ to ease notation. Our proof is constructive. Let us define $u \in \mathbb {R}^n$ as follows:

$$\begin{aligned} u_i = \left\{ \begin{array}{ll} x_i + \lambda (1 - x_i)\,, &  \text {if}~ x_i > 0\,,\\ 0\,, &  \text {otherwise,} \end{array}\right. \end{aligned}$$

where

36 $$\begin{aligned} \lambda := \frac{\rho - 1}{\nu - 1} \in (0,1). \end{aligned}$$

Note that $0 \le x \le u \le e$ and $e^\top u = \rho$. Let us define $X = x x^\top$, $R = x u^\top$, and

37 $$\begin{aligned} U = u u^\top + U^1 + U^2\,, \end{aligned}$$

where

$$\begin{aligned} U^1:= &   \alpha \left( {{\,\textrm{Diag}\,}}( x) - x x^\top \right) ,\\ U^2:= &   \beta \left( {{\,\textrm{Diag}\,}}(a) - a a^\top \right) , \end{aligned}$$

and $\alpha$, $\beta$, and $a \in \mathbb {R}^n$ are given by

38 $$\begin{aligned} \alpha:= &   \frac{(\nu - \rho )(\nu - \rho - 1)}{(\nu - 1)(\nu - 2)} \ge 0, \end{aligned}$$

39 $$\begin{aligned} \beta:= &   \frac{(\nu - \rho )(\rho - 1)}{\nu - 2} > 0, \end{aligned}$$

40 $$\begin{aligned} a_i:= &   {\left\{ \begin{array}{ll} \frac{1 - x_i}{\nu - 1}, &  \text {if}~ x_i > 0, \\ 0, &  \text {otherwise.} \end{array}\right. } \end{aligned}$$

Note that $a \in \mathbb {R}^n_+$, $e^\top a = 1$, and $U e = \rho u$, because $U^1 e =U^2 e=0$. The full matrix

$$\begin{aligned} \begin{bmatrix} 1 & x^\top & u^\top \\ x & X &  R \\ u &  R^\top &  U \end{bmatrix} = \begin{bmatrix} 1\\ x \\ u \end{bmatrix} \begin{bmatrix} 1\\ x \\ u \end{bmatrix}^\top + \begin{bmatrix} 0 &  \quad 0 & \quad 0 \\ 0 &  \quad 0 & \quad 0 \\ 0 &  \quad 0 &  \quad U^1 + U^2 \end{bmatrix} \end{aligned}$$

is positive-semidefinite since $$U^1$$ and $$U^2$$ are so by Lemma 4.4. In addition, if $$ x_i = 0$$, then $$ U_{ii} = 0 = u_i$$. If $$ x_i > 0$$, then it follows as well that $$U_{ii}=u_i$$, along the following lines:

41 $$\begin{aligned} \begin{array}{rcl} U_{ii} &  = &  u_i^2 + \alpha ( x_i - x_i^2) + \beta (a_i - a_i^2) \\ &  = &  \left( (1 - \lambda ) x_i + \lambda \right) ^2 + \alpha ( x_i - x_i^2) + \frac{\beta }{\nu - 1} (1 - x_i) - \frac{\beta }{(\nu - 1)^2} (1 - x_i)^2\\ &  = &  \left( (1 - \lambda )^2 - \alpha - \frac{\beta }{(\nu - 1)^2}\right) x_i^2 + \left( 2 \lambda (1 - \lambda ) + \alpha - \frac{\beta }{\nu - 1} + \frac{2 \beta }{(\nu - 1)^2}\right) x_i \\ &  &  \quad + \lambda ^2 + \frac{\beta }{\nu - 1} - \frac{\beta }{(\nu - 1)^2} \,.\end{array} \end{aligned}$$

We claim the last expression of (41) equals $(1 - \lambda ) x_i + \lambda = u_i$, which follows by equating the coefficients of $x_i^2$, $x_i$, and 1, in above expression and re-arranging all terms with $\lambda$ to the right-hand side:

42 $$\begin{aligned} \left\{ \begin{array}{rcccl} \alpha + \frac{\beta }{(\nu - 1)^2} &  = &  (1 - \lambda )^2 &  = &  \frac{(\nu - \rho )^2}{(\nu - 1)^2}\\ \alpha + \frac{\beta (3-\nu )}{(\nu - 1)^2} &  = & (1 - \lambda ) (1- 2 \lambda ) &  = &  \frac{(\nu - \rho )(\nu - 2 \rho + 1)}{(\nu - 1)^2} \\ 0 + \frac{\beta (\nu -2)}{(\nu - 1)^2} & =&  \lambda (1-\lambda ) &  = &  \frac{(\nu - \rho )(\rho - 1)}{(\nu - 1)^2} \end{array} \right\} \,. \end{aligned}$$

Observe that the system (42) has a unique solution given by (38) and (39) since subtracting the second equation from the first one yields the third equation. Therefore, we obtain that $${{\,\textrm{diag}\,}}( U) = u$$. We clearly have $$ X \ge 0$$ and $$ X - R^\top = ( x - u) x^\top \le 0$$. Finally, we focus on $$ X - R - R^\top + U \ge 0$$. If $$ x_i = 0$$, then $$ U_{ii} - 2 R_{ii} + X_{ii} = 0 \ge 0$$. On the other hand, if $$ x_i > 0$$, we have

$$\begin{aligned} U_{ii} - 2 R_{ii} + X_{ii} = u_i^2 + U^1_{ii} + U^2_{ii} - 2 x_i u_i + x_i^2 = ( u_i - x_i)^2 + U^1_{ii} + U^2_{ii} \ge 0, \end{aligned}$$

where we used $$U^1 \succeq 0$$ and $$U^2 \succeq 0$$. Similarly, $$ U_{ij} - R_{ij} - R_{ji} + X_{ij} = 0 \ge 0$$ whenever $$1 \le i < j \le n$$ and $$ x_i x_j = 0$$. On the other hand, if $$1 \le i < j \le n$$ and $$ x_i x_j > 0$$, we obtain

$$\begin{aligned} U_{ij} - R_{ij} - R_{ji} + X_{ij}= &   u_i u_j - \alpha x_i x_j - \beta a_i a_j - x_i u_j - x_j u_i + x_i x_j \\= &   ( u_i - x_i)( u_j - x_j) - \alpha x_i x_j - \beta a_i a_j \\= &   \lambda ^2 (1 - x_i) (1 - x_j) - \alpha x_i x_j - {\textstyle \frac{\beta }{(\nu - 1)^2}}\, (1 - x_i) (1 - x_j) \\= &   \left( \lambda ^2 - {\textstyle \frac{\beta }{(\nu - 1)^2}}\right) (1 - x_i - x_j) + \left( \lambda ^2 - {\textstyle \frac{\beta }{(\nu - 1)^2}} - \alpha \right) x_i x_j\\= &   {\textstyle \frac{(\rho - 1)(\rho - 2)}{(\nu - 1)(\nu - 2)}}\left( 1 - x_i - x_j\right) + {\textstyle \frac{ 2 \rho - 1 - \nu }{\nu - 1}} \, x_i x_j , \end{aligned}$$

where we used (36), (38), and (39) to derive the last equation. Since $$\rho \ge 2$$, $$\nu \ge 3$$, $$1 - x_i - x_j\ge 0$$ and $$ x_i x_j > 0$$, it follows that $$ U_{ij} - R_{ij} - R_{ji} + X_{ij} \ge 0$$ if $$\nu \le 2 \rho - 1$$, which establishes (32) and (33). If, on the other hand, $$\nu > 2 \rho - 1$$, then $$ U_{ij} - R_{ij} - R_{ji} + X_{ij} \ge 0$$ by (35), giving rise to (34). This completes the proof. $$\square $$

#### Remark 4.2

As pointed out by a reviewer, the construction of the matrix *U* given by (37) in the proof of Theorem 4.3 ensures that $U \succeq 0$. Furthermore, we have $U \ge 0$ by Lemma 3.1, which implies that *U* is doubly nonnegative. It follows from [17] that *U* is completely positive if $\nu = \Vert x\Vert _0 \le 4$. An interesting question raised by the reviewer is whether *U* is completely positive even if $\nu = \Vert x\Vert _0 \ge 5$. Our result below gives a partial answer.

#### Lemma 4.8

Let $\rho \in \{1,\ldots ,n\}$ and let $x \in F$ be such that $\nu = \Vert x\Vert _0 \ge \rho + 1$. Let $U \in \mathcal{S}^n$ be given by (37), where *u*, $U^1$, and $U^2$ are defined as in the proof of Theorem 4.3. If

43 $$\begin{aligned} 2\rho \le \nu \le \rho + \frac{\rho -2}{x^\top x}, \end{aligned}$$

then *U* is completely positive.

#### Proof

First, we observe that the upper bound on $\nu$ in (43) is at least as large as the lower bound if and only if $x^\top x \le 1 - \frac{2}{\rho }$, which can be satisfied only if $\rho \ge 3$ since $x \in F$. We therefore obtain $\nu \ge 6$ by the first inequality in (43). Furthermore, for $\rho = 3$ and $\nu = 6$, (43) holds for any $x \in F$ such that $x^\top x \le \frac{1}{3}$. Since $x^\top x \ge \frac{1}{\nu }$ for each $x \in F$ such that $\Vert x\Vert _0 = \nu$, we conclude that the set of triples $(\rho , \nu , x)$ that satisfy the hypothesis is nonempty.

Clearly, we only need to prove the assertion for the submatrix $W \in \mathcal{S}^{\nu }$ of *U* that are indexed by the positive indices of *u*. To that end, let us denote the subvector of *x* restricted to its positive entries by $x_{\textbf{P}} \in \mathbb {R}^{\nu }$. Using (37), straightforward but tedious calculations reveal that

$$\begin{aligned} W= &   \frac{(\nu -\rho )(\rho -1)}{(\nu -1)(\nu -2)} \left[ \frac{ \rho - 2 }{\nu - \rho }\, e e^\top + x_{\textbf{P}} \, e^\top + e \, x_{\textbf{P}}^\top + I + \frac{ \nu - 2\rho }{ \rho - 1 } {{\,\textrm{Diag}\,}}(x_{\textbf{P}}) \right] \\= &   \frac{(\nu -\rho )(\rho -1)}{(\nu -1)(\nu -2)} \left[ (e \, x_{\textbf{P}}^\top + I)(e \, x_{\textbf{P}}^\top + I)^\top \right. \\  &   \left. + \frac{ \rho - 2 - (x^\top x)(\nu - \rho ) }{\nu - \rho }\, e e^\top + \frac{ \nu - 2\rho }{\rho - 1} \, {{\,\textrm{Diag}\,}}(x_{\textbf{P}}) \right] , \end{aligned}$$

where $e \in \mathbb {R}^{\nu }$, $I \in \mathcal{S}^{\nu }$, and we used $x_{\textbf{P}}^\top \, x_{\textbf{P}} = x^\top x$ in the second equality. Using $\rho \ge 3$, $\nu \ge \rho + 1$ and (43), we conclude that $W \in \mathcal{S}^{\nu }$ is a completely positive matrix as it is given by the sum of nonnegative combinations of three completely positive matrices $(e \, x_{\textbf{P}}^\top + I)(e \, x_{\textbf{P}}^\top + I)^\top$, $e e^\top$, and ${{\,\textrm{Diag}\,}}(x_{\textbf{P}}) = \sum \limits _{i: x_i > 0} x_i e^i (e^i)^\top$. This completes the proof. $\square$

Lemma 4.8 establishes the stronger result that the matrix *U* given by (37) is completely positive under additional assumptions. We remark, however, that this result does not necessarily extend to the full matrix in the proof of Theorem 4.3.

Before we proceed to the important consequences of Theorem 4.3, let us motivate the construction in its proof, in particular the choice of $\lambda$ and the other constants.

#### Observation 4.1

Let $$\rho \in \{ 2,\ldots ,n\}$$ and let $${x}\in F$$. Assume that $$ {u}_i = \tau {x}_i +b$$ if $$x_i>0$$ with $$0<\tau <1$$, while $$u_i=0$$ if $$x_i=0$$. Furthermore, assume that $$ {U}_{ij} = c {x}_i + c {x}_j +d$$ if $$x_ix_j>0$$ while $$U_{ij}=0$$ if $$x_ix_j=0$$ for $$1 \le i < j \le n$$. It is easy to verify that the choices of *u* and *U* in the proof of Theorem 4.3 are in this form. Then, the best choice of $$\tau $$, *b*, *c* and *d* ensuring that $$(x, u, X, U, R) = ( {x}, {u}, {x} {x}^\top , {U}, {x} {u}^\top )$$ is (R3($$\rho $$))-feasible, is the choice in the proof of Theorem 4.3.

#### Proof

Let $\rho \in \{ 2,\ldots ,n\}$ and let ${x}\in F$. Again, abbreviate $\nu =\Vert {x}\Vert _0$. From $e^\top (\tau {x} +b) = e^\top u= \rho$, we derive $b = \frac{\rho -\tau }{\nu }\in (0,1)$ as $\rho>1>\tau$ and $\rho -\tau< \rho < \nu$. Furthermore, the constraints $x\le u \le e$ become

$$\begin{aligned} x_i \le \min \left\{ {\frac{1-b}{\tau }, \frac{b}{1-\tau }}\right\} = \min \left\{ {\frac{ \nu -\rho +\tau }{ \nu \tau }, \frac{\rho -\tau }{ \nu (1-\tau )}}\right\} \quad \text{ for } \text{ all } i = 1,\ldots ,n\,. \end{aligned}$$

Since $g(\tau ):=\frac{ \nu -\rho +\tau }{ \nu \tau }$ decreases and $h(\tau ):=\frac{\rho -\tau }{ \nu (1-\tau )}$ increases with $\tau \in (0,1)$, the maximum of $\min \left\{ g(\tau ), h(\tau )\right\}$ is attained at $\tau ^*$ satisfying $g(\tau ^*)=h(\tau ^*)$, and this value ensures that the formulation covers as many $x\in F$ as possible. Hence the best choice of $\tau$ would be the solution $\tau ^*$ of $g(\tau ^*)=h(\tau ^*)$, namely $\tau ^*=\frac{ \nu -\rho }{ \nu -1}$, which is exactly our choice in the proof of Theorem 4.3 with $\lambda = 1 - \tau ^*= \frac{\rho -1}{\nu -\rho }$. Since $U_{ii} = u_i$ and $Ue = \rho u$, we have for $x_i>0$

$$\begin{aligned} \begin{aligned} \tau ^* x _i + b + \sum _{j\ne i:x_j>0}(c x _i + c x _j +d)&=\rho (\tau ^* x _i + b)\quad \text{ or } \\ ( \nu -1)d + c + \frac{\rho - \tau ^*}{ \nu } + ( \nu -1)c x _i + (\tau ^* - c) x _i&= \rho \tau ^* x _i + \frac{\rho (\rho - \tau ^*)}{ \nu }, \end{aligned} \end{aligned}$$

which implies, comparing coefficients of $x_i$ and 1, that

$$\begin{aligned}  &   ( \nu -1)c + \tau ^* - c = \rho \tau ^* \quad \text{ and }\\  &   ( \nu -1)d + c + \frac{\rho - \tau ^*}{ \nu } = \frac{\rho (\rho - \tau ^*)}{ \nu } \,, \end{aligned}$$

so that $c = \frac{(\rho -1)\tau ^*}{ \nu -2}= \frac{(\rho -1)(\nu -\rho )}{(\nu -1)(\nu -2)}$ and $d = \frac{\rho -1}{\nu (\nu -1)(\nu -2)}[(\nu -2)\rho -2\tau ^*( \nu -1)] =\frac{(\rho -1)(\rho -2)}{ ( \nu -1)( \nu -2)}$, substituting $\tau ^* = \frac{ \nu -\rho }{ \nu -1}$. This justifies our choice of *c* and *d* in the proof of Theorem 4.3. $\square$

#### Example 4.1

The condition (35) is sufficient but not necessary. For $n = 6$ and $\rho = 3$, the point

$$\begin{aligned} x = [0.6,0.2,0.05,0.05,0.05,0.05]^\top \in F \end{aligned}$$

violates (35) since

$$\begin{aligned} 0.6= &   \frac{(0.6)\, (0.2)}{1 - 0.6 - 0.2} = \max \limits _{1 \le i < j \le n: x_i x_j> 0} \frac{x_i x_j}{1 - x_i - x_j}\\> &   \frac{(\rho - 1)(\rho - 2)}{\left( \Vert x\Vert _0 - 2\right) \left( \Vert x\Vert _0 - 2 \rho + 1\right) } = 0.5, \end{aligned}$$

while there exists $(u, U, R) \in \mathbb {R}^6 \times \mathcal{S}^6 \times \mathbb {R}^{6 \times 6}$ such that $(x, u, X, U, R) = ( {x}, {u}, {x} {x}^\top , {U}, {x} {u}^\top )$ is (SDP-RLT(3))-feasible. One choice of *u* and *U* is $u = [0.8866,0.5512,0.3905,0.3906,0.3906,0.3905]^\top$ and

$$\begin{aligned} U = \begin{bmatrix} 0.8866 & 0.4674 & 0.3264 & 0.3265 & 0.3265 & 0.3264\\ 0.4674 & 0.5512 & 0.1588 & 0.1588 & 0.1588 & 0.1588\\ 0.3264 & 0.1588 & 0.3905 & 0.0986 & 0.0986 & 0.0986\\ 0.3265 & 0.1588 & 0.0986 & 0.3906 & 0.0986 & 0.0986\\ 0.3265 & 0.1588 & 0.0986 & 0.0986 & 0.3906 & 0.0986\\ 0.3264 & 0.1588 & 0.0986 & 0.0986 & 0.0986 & 0.3905\\ \end{bmatrix}. \end{aligned}$$

Note that *u* is not given by an affine function of *x* in the sense of Observation 4.1.

Theorem 4.3 reveals that an increasingly larger and nontrivial set of rank-one solutions is contained in the sets $\mathcal{F}^{R3}_\rho$ as $\rho$ increases. Note that $G_\rho$ given by (35) is a nonconvex set. Our next result gives further insight into this set by providing a piecewise convex inner approximation.

#### Lemma 4.9

We have $G_2 = \emptyset$. Furthermore, for all $\rho \in \left\{ 3,\ldots ,\left\lfloor \textstyle {\frac{n}{2}} \right\rfloor \right\}$, define

44 $$\begin{aligned} \delta _{\rho ,\nu }:= 2 \left[ \left( \tau _{\rho ,\nu }^2 + \tau _{\rho ,\nu }\right) ^{1/2} - \tau _{\rho ,\nu }\right] , \end{aligned}$$

with

45 $$\begin{aligned} \tau _{\rho ,\nu }:= \frac{(\rho - 1)(\rho - 2)}{\left( \nu - 2\right) \left( \nu - 2 \rho + 1\right) }\,. \end{aligned}$$

Then,

46 $$\begin{aligned} H_\rho := \bigcup \limits _{\nu = 2 \rho }^n \left\{ x \in F: \Vert x \Vert _0 = \nu , \quad x_i + x_j \le \delta _{\rho ,\nu },~1 \le i < j \le n\right\} \subseteq G_\rho \,, \end{aligned}$$

where $G_\rho$ is defined as in (35). Moreover, we have

47 $$\begin{aligned} \left\{ (x, x x^\top ) \in \mathcal{F}^{R3}: x \in H_\rho \right\} \subseteq \mathcal{F}^{R3}_\rho \qquad \text{ for } \text{ all } \rho \in \left\{ 3, \ldots , \left\lfloor \textstyle {\frac{n}{2}} \right\rfloor \right\} \,. \end{aligned}$$

#### Proof

For $\rho = 2$, the upper bound in (35) equals zero, which implies that $G_2 = \emptyset$. Let us fix $\rho \in \left\{ 3,\ldots ,\left\lfloor \textstyle {\frac{n}{2}} \right\rfloor \right\}$ and let $x \in H_\rho$. Then, $x \in F$, $\Vert x \Vert _0 = \nu > 2 \rho - 1$, and it is easy to verify that

$$\begin{aligned} \max \limits _{1 \le i < j \le n: x_i x_j > 0} \frac{x_i x_j}{1 - x_i - x_j} \le \frac{\delta _{\rho ,\nu }^2}{4 \left( 1 - \delta _{\rho ,\nu } \right) } = \tau _{\rho ,\nu } \,, \end{aligned}$$

where the last equality follows from (44) and (45). Both inclusions (46) and (47) now follow from Theorem 4.3 by observing that $\Vert x \Vert _0 = \nu$. $\square$

For fixed $\rho \in \left\{ 3,\ldots ,\left\lfloor \textstyle {\frac{n}{2}} \right\rfloor \right\}$, it is worth noticing that $\tau _{\rho ,\nu }$ given by (45) is a decreasing function of $\nu$, which, in turn, implies that $\delta _{\rho ,\nu }$ given by (44) is a decreasing function of $\nu$. Therefore, the positive components of the elements of $H_\rho$ given by Lemma 4.9 tend to get closer to each other as $\nu$ increases. For instance, if $\rho = 3$, then $\delta _{\rho ,\nu }$ equals 0.7321, 0.5798, and 0.4805 for $\nu = 6$, $\nu = 7$, and $\nu = 8$, respectively. Note that the point *x* of Example 4.1 satisfies $x\notin G_3$, readily certifying $x\notin H_3$ since $x_1+x_2=0.8>0.7321$.

Theorem 4.3 gives rise to several results about rank-one solutions of $\mathcal{F}^{R3}_\rho$. Our next result gives a complete description of such solutions for $\rho = 2$.

#### Corollary 4.6

We have $( x, x x^\top ) \in \mathcal{F}^{R3}_2$ if and only if $x \in F_3$.

#### Proof

By Theorem 4.3, for any $x \in F_3$, we have $( x, x x^\top ) \in \mathcal{F}^{R3}_2$ by (32). The assertion follows from Corollary 4.5. $\square$

For $\rho = 1$ and $\rho = 2$, it follows from Corollary 4.4 and Corollary 4.6 that $( x, x x^\top ) \in \mathcal{F}^{R3}_\rho$ if and only if $x \in F_1$ and $x \in F_3$, respectively. On the other hand, for $\rho \ge 3$, Theorem 4.3 gives rise to our next result, which reveals that such a nontrivial upper bound on $\Vert x \Vert _0$ concerning rank-one solutions of $\mathcal{F}^{R3}_\rho$ does not exist.

#### Lemma 4.10

Let $\rho \in \{3,\ldots ,n-1\}$. Then, for any $\nu \in \{\rho + 1,\ldots ,n\}$, there exists $x \in F_{\nu }$ such that $( x, x x^\top ) \in \mathcal{F}^{R3}_\rho$.

#### Proof

Let $\rho \in \{3,\ldots ,n-1\}$ and $\nu \in \{\rho + 1,\ldots ,n\}$. By Theorem 4.3, the assertion clearly holds for any $x \in F_{\nu }$ such that $\Vert x \Vert _0 = \nu \le 2 \rho - 1$. Suppose that $\nu > 2 \rho - 1$. By Lemma 4.9, it suffices to construct an $x \in F_{\nu }$ such that $\Vert x \Vert _0 = \nu$ and $x \in H_\rho$, where $H_\rho$ is defined as in (46). Let $x \in F_{\nu }$ be given by

$$\begin{aligned} x_i = \left\{ \begin{array}{ll} \frac{1}{\nu }, &  \text {if } i \in \{ 1,\ldots ,\nu \}\,, \\ 0\,, &  \text {otherwise.} \end{array}\right. \end{aligned}$$

We therefore need to verify that

$$\begin{aligned} \frac{2}{\nu } \le 2 \left[ \left( \tau _{\rho ,\nu }^2 + \tau _{\rho ,\nu }\right) ^{1/2} - \tau _{\rho ,\nu }\right] \,, \end{aligned}$$

where $\tau _{\rho ,\nu }$ is given by (45). Rearranging and simplifying the terms, the above inequality reduces to

$$\begin{aligned} \frac{1}{\nu (\nu - 2)} \le \tau _{\rho ,\nu }\,. \end{aligned}$$

By (45), this inequality holds if

$$\begin{aligned} \frac{\nu - 2 \rho + 1}{\nu } \le (\rho - 1)(\rho - 2)\,. \end{aligned}$$

Since $\rho \ge 3$ (and thus $2\rho -1>0$), we even have

$$\begin{aligned} \frac{\nu - 2 \rho + 1}{\nu } \le 1 \le (\rho - 1)(\rho - 2)\,, \end{aligned}$$

which establishes the assertion. $\square$

Following our earlier discussion about the positive components of the elements of the set $H_\rho$, we remark that all such components of the solution constructed in the proof of Lemma 4.10 are equal. Our next result establishes another useful property of the rank-one solutions of $\mathcal{F}^{R3}_\rho$.

#### Theorem 4.4

For each $\rho \in \{1,\ldots ,n-1\}$, if $(x, xx^\top ) \in \mathcal{F}^{R3}_\rho$, then $(x, xx^\top ) \in \mathcal{F}^{R3}_{\rho +1}$.

#### Proof

If $\rho = 1$, then the claim follows from Corollary 4.4 and Corollary 4.6. Therefore, let $\rho \in \{{2},\ldots ,n-1\}$ and let $(x, xx^\top ) \in \mathcal{F}^{R3}_\rho$. Let us define $\nu = \Vert x \Vert _0$. If $\rho \in \left\{ 2,\ldots ,\left\lfloor \textstyle {\frac{n+1}{2}}\right\rfloor - 1\right\}$ and $\nu \le 2 (\rho + 1) - 1 = 2 \rho + 1$; or if $\rho \in \left\{ \left\lfloor \textstyle {\frac{n+1}{2}} \right\rfloor , \ldots ,n\right\}$, then the assertion follows from Theorem 4.3. Therefore, let us assume that $\rho \in \left\{ 2,\ldots ,\left\lfloor \textstyle {\frac{n+1}{2}}\right\rfloor - 1\right\}$ and $\nu > 2 \rho + 1$. For each $\rho \ge 3$, we remark that the set of rank-one solutions with this property is nonempty by Lemma 4.10.

Since $(x, xx^\top ) \in \mathcal{F}^{R3}_\rho$, there exists $(u,U,R) \in \mathbb {R}^n \times \mathcal{S}^n \times \mathbb {R}^{n \times n}$ such that $( x, u, x x^\top , U, R) \in \mathbb {R}^n \times \mathbb {R}^n \times \mathcal{S}^n \times \mathcal{S}^n \times \mathbb {R}^{n\times n}$ is (R3($\rho$))-feasible. Since $X = x x^\top$, we have $R = x u^\top$ and $U - u u^\top \succeq 0$ by the Schur complement lemma. We will construct $(u^\prime , U^\prime , R^\prime ) \in \mathbb {R}^n \times \mathcal{S}^n \times \mathbb {R}^{n \times n}$ such that $( x, u^\prime , x x^\top , U^\prime , R^\prime ) \in \mathbb {R}^n \times \mathbb {R}^n \times \mathcal{S}^n \times \mathcal{S}^n \times \mathbb {R}^{n\times n}$ is (R3($\rho + 1$))-feasible. To this end, given *u* with $I_u:=\{i: u_i >0\}$, we construct a convex combination $u'= (1-\lambda )u + \lambda e(u)$ where $e(u):= \sum \limits _{i\in I_u} e_i$ and $\lambda := \frac{1}{\mu }$ where

$$\begin{aligned} \mu := \Vert u \Vert _0 - \rho > \rho + 1 \ge 3\,, \end{aligned}$$

by assumption on $\Vert u \Vert _0 \ge {\Vert x \Vert }_0=\nu > 2\rho +1$. Therefore, $u^\prime = u + s$, where $s \in \mathbb {R}^n_+$ is given by

$$\begin{aligned} s_i = \left\{ \begin{array}{ll} \frac{1 - u_{i}}{\Vert u \Vert _0 - \rho }, &  \hbox { if}\ u_i > 0,\\ 0, &  \text {otherwise.} \end{array}\right. \end{aligned}$$

The inequality $s_i \ge 0$ follows from $u_i\le 1$. Therefore, we obtain $0 \le x \le u \le u^\prime \le e$. Furthermore, $e^\top s = 1$, which implies that $e^\top u^\prime = \rho + 1$. Since $X = x x^\top$, we define $R^\prime = x (u^\prime ){^\top } = R + x s^\top$. Finally, we define

$$\begin{aligned} U^\prime = u^\prime (u^\prime ){^\top } + \frac{\mu - 2}{\mu } \left( U - uu{^\top } \right) + {{\,\textrm{Diag}\,}}(s) - ss{^\top } \,. \end{aligned}$$

By the Schur complement lemma, we have

$$\begin{aligned} \begin{bmatrix} X &  R^\prime \\ (R^\prime )^\top &  U^\prime \end{bmatrix}  - \begin{bmatrix} x \\ u^\prime \end{bmatrix} \begin{bmatrix} x \\ u^\prime \end{bmatrix} ^\top = \begin{bmatrix} 0 &  \quad 0 \\ 0 &  \quad \frac{\mu - 2}{\mu } \left( U - uu{^\top } \right) + {{\,\textrm{Diag}\,}}(s) - ss{^\top } \end{bmatrix} \succeq 0\,, \end{aligned}$$

where we used $U - u u^\top \succeq 0$, $\mu > 3$, and Lemma 4.4 for ${{\,\textrm{Diag}\,}}(s) - ss^\top \succeq 0$. Therefore, the semidefiniteness constraint is satisfied. We clearly have $X e = x$, $R^\prime e = (\rho + 1) x$, $(R^\prime )^\top e = u^\prime$, and $U^\prime e = (\rho + 1) u^\prime$. We next focus on the constraint ${{\,\textrm{diag}\,}}(U^\prime ) = u^\prime$. If $u_i = 0$, then $u^\prime _i = u_i = U_{ii} = U^\prime _{ii} = 0$ since $s_i = 0$. If $u_i > 0$, then

$$\begin{aligned} U^\prime _{ii}= &   (u^\prime _i)^2 + \frac{\mu - 2}{\mu } \left( U_{ii} - u_i^2 \right) + s_i - s_i^2 \\= &   \left( u_i + s_i \right) ^2 + \frac{\mu - 2}{\mu } \left( u_i - u_i^2 \right) + s_i - s_i^2 \\= &   \frac{2}{\mu } u_i^2 + \frac{\mu - 2}{\mu } u_i + s_i + 2 u_i s_i \\= &   \frac{1}{\mu } \left( 2 u_i^2 + (\mu - 2) u_i + 1 - u_i + 2 u_i (1 - u_i) \right) \\= &   \frac{(\mu - 1) u_i + 1}{\mu } \\= &   u^\prime _i, \end{aligned}$$

where we used ${{\,\textrm{diag}\,}}(U) = u$ in the second line and the definition of *s* in the fourth line. This establishes ${{\,\textrm{diag}\,}}(U^\prime ) = u^\prime$.

Furthermore, we have $X \ge 0$, $X - (R^\prime )^\top = X - R^\top - s x^\top \le 0$ since $X - R^\top \le 0$, $x \ge 0$, and $s \ge 0$. We next verify $X - (R^\prime )^\top - R^\prime + U^\prime \ge 0$. For the diagonal components, we have

$$\begin{aligned} U^\prime _{ii} - 2 R^\prime _{ii} + X_{ii}= &   u^\prime _i - 2 x_i u^\prime _i + x_i^2 \ge (u^\prime _i)^2 \\    &   - 2 x_i u^\prime _i + x_i^2 = ( u_i{^\prime } - x_i)^2 \ge 0, \quad i = 1,\ldots ,n, \end{aligned}$$

where we used ${{\,\textrm{diag}\,}}(U^\prime ) = u^\prime$ and $0 \le x \le u \le e$. If $1 \le i < j \le n$, then

$$\begin{aligned} U^\prime _{ij} - R^\prime _{ij} - R^\prime _{ji} + X_{ij}= &   u^\prime _i u^\prime _j + \frac{\mu - 2}{\mu } \left( U_{ij} - u_i u_j \right) - s_i s_j - x_i u^\prime _j - x_j u^\prime _i + x_i x_j \\= &   (u_i + s_i) (u_j + s_j) + \frac{\mu - 2}{\mu } \left( U_{ij} - u_i u_j \right) - s_i s_j \\  &   \quad - x_i (u_j + s_j) - x_j (u_i + s_i) + x_i x_j \\= &   u_i s_j + s_i u_j - x_i u_j - x_i s_j - x_j u_i - x_j s_i + x_i x_j \\  &   \quad + \frac{2}{\mu } u_i u_j + \frac{\mu - 2}{\mu } U_{ij} \\= &   \frac{\mu - 2}{\mu } \left( U_{ij} - x_i u_j - x_j u_i + x_i x_j \right) \\  &   \quad + \frac{2}{\mu } \left( u_i u_j - x_i u_j - x_j u_i + x_i x_j \right) \\  &   \quad + u_i s_j + s_i u_j - x_i s_j - x_j s_i \\\ge &   \frac{2}{\mu } \left( (u_i - x_i) (u_j - x_j) \right) + \left( s_j (u_i - x_i) + s_i (u_j - x_j) \right) \\\ge &   0, \end{aligned}$$

where we used $\mu > 3$ and $U_{ij} - R_{ij} - R_{ji} + X_{ij} = U_{ij} - x_i u_j - x_j u_i + x_i x_j \ge 0$ to derive the first inequality, and $0 \le x \le u$ together with $s \ge 0$ to arrive at the final one. This completes the proof. $\square$

Theorem 4.4 establishes the nested behavior of the sets of rank-one solutions of $\mathcal{F}^{R3}_\rho$ with respect to $\rho$. We close this section with the following result about the tightness of the lower bound $\ell ^{R3}_\rho (Q)$ arising from (R3).

#### Corollary 4.7

We have

48 $$\begin{aligned} \ell ^{R3}_\rho (Q)\le &   \ell _{2 \rho - 1}(Q) {\, \le \ell _{\rho }(Q)} , \quad \text {\quad {for all} } \rho \in \left\{ 2, \ldots , \left\lfloor \textstyle {\frac{n+1}{2}} \right\rfloor \right\} , \text { while} \end{aligned}$$

49 $$\begin{aligned} \ell ^{R3}_\rho (Q)\le &   \ell (Q) {\, \quad \quad \le \ell _{\rho }(Q)}, \quad \text {\quad {for all} }\rho \in \left\{ \left\lfloor \textstyle {\frac{n+1}{2}} \right\rfloor + 1, \ldots ,n\right\} . \end{aligned}$$

#### Proof

The first inequalities follow from (22) and from (32) and (33), respectively, whereas the second inequalities follow from Lemma 2.1. $\square$

Corollary 4.7 reveals that the lower bound $\ell ^{R3}_\rho (Q)$ can be potentially quite weak especially for larger values of $\rho$.

## Concluding Remarks

A Standard Quadratic optimization Problem with hard sparsity constraints can be exactly reformulated as a mixed-binary QP. Therefore, it is tempting to use tractable LP- or SDP-based relaxations, either in a straightforward way or by suitable combinations as we did in Sect. 4. The aim is to achieve tight rigorous bounds with a computational effort that scales well with the problem size.

We characterized the exactness of the bounds and studied the behavior of rank-one solutions to the relaxations. However, our analysis reveals that some caveats are in place when following this approach. In unfavorable circumstances (e.g., if the sparsity constraints are not stringent enough), the resulting bounds are quite weak.

Note that replacing the constraint $e^\top u = \rho$ in (StQP($\rho$)) with $e^\top u \le \rho$ gives rise to an alternative formulation. In a similar manner, one can construct the corresponding LP- and SDP-based relaxations of this variant. It is easy to show that the resulting convex relaxations cannot be stronger than their counterparts considered in this study. However, our preliminary computational experiments suggest that the lower bounds arising from the relaxations of both versions agree. A theoretical investigation of this observation is left as a future research direction.

The findings of this article definitely call for more investigation, either in the direction of refined RLT models, or equally importantly, tighter conic-based relaxations which still offer some tractability (see [9] for some recent progress on the second point). While these avenues are beyond the scope of the present work, they remain on our research agenda for the near future.

## Data Availability

The manuscript has no associated data.
